# Assessing the Dynamic Outcomes of Containment Strategies against COVID-19 under Different Public Health Governance Structures: A Comparison between Pakistan and Bangladesh

**DOI:** 10.3390/ijerph19159239

**Published:** 2022-07-28

**Authors:** Weiwei Zhang, Thomas Huggins, Wenwen Zheng, Shiyong Liu, Zhanwei Du, Hongli Zhu, Ahmad Raza, Ahmad Hussen Tareq

**Affiliations:** 1 Research Institute of Economics and Management, Southwestern University of Finance and Economics, Chengdu 610074, China; weiweizhang@smail.swufe.edu.cn (W.Z.); honglizhu9311@gmail.com (H.Z.); ahmadraza_1@hotmail.com (A.R.); 2 Division of Science & Technology, BNU-HKBU United International College, Zhuhai 519087, China; tjhuggins@uic.edu.cn; 3 Personal Finance Department, HQ of China Construction Bank, Beijing 100033, China; lilyzheng69@163.com; 4 Institute of Advanced Studies in Humanities and Social Sciences, Beijing Normal University at Zhuhai, Zhuhai 519087, China; 5 Division of Epidemiology and Biostatistics, School of Public Health, Hong Kong University, Hong Kong, China; zwdu@hku.hk; 6 Ministry of National Health Services Regulations and Coordination, Islamabad 44010, Pakistan; drahmad@hsa.edu.pk; 7 Health Services Academy, Islamabad 44010, Pakistan

**Keywords:** COVID-19, Pakistan, Bangladesh, containment strategies, counterfactual analysis

## Abstract

COVID-19 scenarios were run using an epidemiological mathematical model (system dynamics model) and counterfactual analysis to simulate the impacts of different control and containment measures on cumulative infections and deaths in Bangladesh and Pakistan. The simulations were based on national-level data concerning vaccination level, hospital capacity, and other factors, from the World Health Organization, the World Bank, and the Our World in Data web portal. These data were added to cumulative infections and death data from government agencies covering the period from 18 March 2020 to 28 February 2022. Baseline curves for Pakistan and Bangladesh were obtained using piecewise fitting with a consideration of different events against the reported data and allowing for less than 5% random errors in cumulative infections and deaths. The results indicate that Bangladesh could have achieved more reductions in each key outcome measure by shifting its initial lockdown at least five days backward, while Pakistan would have needed to extend its lockdown to achieve comparable improvements. Bangladesh’s second lockdown appears to have been better timed than Pakistan’s. There were potential benefits from starting the third lockdown two weeks earlier for Bangladesh and from combining this with the fourth lockdown or canceling the fourth lockdown altogether. Adding a two-week lockdown at the beginning of the upward slope of the second wave could have led to a more than 40 percent reduction in cumulative infections and a 35 percent reduction in cumulative deaths for both countries. However, Bangladesh’s reductions were more sensitive to the duration of the lockdown. Pakistan’s response was more constrained by medical resources, while Bangladesh’s outcomes were more sensitive to both vaccination timing and capacities. More benefits were lost when combining multiple scenarios for Bangladesh compared to the same combinations in Pakistan. Clearly, cumulative infections and deaths could have been highly impacted by adjusting the control and containment measures in both national settings. However, COVID-19 outcomes were more sensitive to adjustment interventions for the Bangladesh context. Disaggregated analyses, using a wider range of factors, may reveal several sub-national dynamics. Nonetheless, the current research demonstrates the relevance of lockdown timing adjustments and discrete adjustments to several other control and containment measures.

## 1. Introduction

As of 19 April 2022, the COVID-19 pandemic caused by SARS-CoV-2 [1] has led to more than 458 million confirmed cases and 6.2 million deaths across almost 200 countries and regions [2]. Since the beginning of the COVID-19 outbreak, SARS-CoV-2 variants have been continually emerging. The Delta variant, which spreads more easily and causes more severe cases than early variants (Alpha, Beta, and Gamma), was first identified in India in late 2020 [3], and new infections soared again around the globe, especially in India. Pakistan and Bangladesh, which surround India, were hit hard in the Spring of 2021. This study compares the dynamic outcomes of COVID-19 containment and control strategies under different public health governance structures using Pakistan and Bangladesh since both South Asian Muslim-majority countries have long shared a similar cultural heritage and were administered as a single state for 24 years under the “One Unit Scheme” program [4,5]. Pakistan and Bangladesh had similar levels of poorly-equipped medical facilities far from the WHO’s minimum standards [6,7,8,9]. The two countries reported their first COVID-19 cases within days of each other (26 February 2020, in Pakistan and 8 March 2020, in Bangladesh). They practically adopted identical testing protocols and clinical diagnosis guidelines, and obtained similar vaccination rates (As of 18 February 2022, only 41.97% and 44.56% of the total population in Pakistan and Bangladesh completed the initial vaccination protocol) [10]. Nonetheless, COVID-19 had divergent impacts on the two countries. With the emergence of the more contagious and less deadly COVID variant-Omicron in South Africa on 24 November 2021 [11], Pakistan and Bangladesh witnessed a surge in infections starting at the end of December 2021, which as of 18 February 2022 had brought the two countries’ total confirmed cases to more than 1.49 and 1.93 million with death tolls of 29,917 and 28,931, respectively [12,13].

With the outbreak of the COVID-19 pandemic, scholars have done extensive research examining the spreading mechanism of COVID-19, exploring effective containment and control strategies, and evaluating the direct and indirect impacts caused by the pandemic [14,15]. System dynamics models (SDMs) or compartmental models have been broadly used to capture the nonlinear dynamics of complex systems over time and integrate the time delays and feedback loops prevalent in disease transmission and progression as well as in public health [16]. SDMs have been widely applied in investigating contagious diseases [17,18] and non-communicable chronic diseases [19,20]. The applications of SDMs have been widely seen in the areas of health service improvement [21,22], impact assessments of policies and interventions [23,24,25,26], resource allocation [25], national health planning [27,28], and the determining the complexities of health-related socioeconomic systems [29,30]. SDMs have also found their extensive applications in the research of the COVID-19 pandemic including (but not limited to) spreading dynamics, trend analysis and prediction, impact assessments of control and containment measures, etc. [31,32,33,34].

In the past two years, researchers have also extensively used SDMs to investigate the COVID-19 spread dynamics in Pakistan and Bangladesh. Kakakhel et al. analyzed COVID-19’s prevalence and epidemiologic trends in Pakistan with an SIR (Susceptible-Infectious-Recovered) model [35]. Ahmad et al. investigated the outbreak of COVID-19 in Pakistan through an SEIR (Susceptible-Exposed-Infectious-Recovered) fractional model [36]. Hussain et al. modeled the dynamic transmission of COVID-19 infection in Pakistan through a deterministic model SEIQR (Susceptible-Exposed-Infectious-Quarantine-Recovered) [37]. Hassan et al. predicted the COVID-19 spread in Bangladesh through an SIR model [38]. Masud et al. used the SEIQR (Susceptible-Exposed-Infectious-Isolated-Recovered) model to understand and discuss the COVID-19 outbreak scenarios in Bangladesh [39]. Akanda et al. examined the pandemic dynamics and the effectiveness of policy measures in Bangladesh by using the SEIRD (Susceptible-Exposed-Infectious-Recovered-Death) model [40]. Truelove et al. used a stochastic SEIR model to simulate COVID-19’s outbreak and spread in refugee camps in Bangladesh and beyond [41]. Kamrujjaman et al. simulated the possible consequences of COVID-19 in the Rohingya camps at Cox’s Bazar in Bangladesh through a modified SEIR transmission model [42]. Khan et al. used a classic SIR model for the prediction and analysis of COVID-19 outbreak in Bangladesh [43].

Beyond studies focused on either a basic susceptible-infectious-recovered (SIR) model or slight extensions, there have been very few studies exploring the impacts of pharmaceutical interventions (PIs), i.e., vaccination, in these contexts. There is also a range of non-pharmaceutical interventions (NPIs), including mask usage lockdowns and hospital (including quarantine hospital and mobile cabin hospital) admissions, which have not been accounted for. To overcome and surpass the limitations of the prior research, the current research uses an SDM that integrates Susceptible, Exposed, Asymptomatic Infections, Symptomatic Infections, Recovered, Death, Hospitalization, and Vaccination (SEASIRD-HV) state variables into a compartmental model (SDM), which simultaneously considers NPIs such as lockdown and hospital capacity and PIs such as vaccination-related factors (vaccination rate and inoculation time).

The resulting model, outlined in Section 2, is used to identify issues and gaps in the COVID-19 containment and control in the two countries. A counterfactual analysis is also outlined, in an effort to identify outcomes that could have been achieved under alternative implementation protocols, and to better understand the interactions between the intervention types. Section 3 provides a summary of the analytical findings. This summary is followed by Section 4, which outlines the implications of the current results for policy design and implementation and for ongoing research.

## 2. Materials and Methods

### 2.1. Design of the Study

As outlined in the Section 1, an evidence-based data-driven SEASIRD-HV system dynamics model (SDM) was built to determine the differences and similarities (if present) of COVID-19 outbreak dynamics under different public health governance structures in Pakistan and Bangladesh. This analysis used the publicly available data sets and statistics outlined below. Once the model had been effectively fit to available data, the analysis proceeded to compare counterfactual scenarios by manipulating key interventions and their cumulative impact on transmission outcomes.

### 2.2. Data Sources

Data concerning COVID-19 cases in Pakistan and Bangladesh were extracted from the Ministry of National Health Services Regulations and Coordination of Pakistan; Institute of Epidemiology, Disease Control, and Research of Bangladesh; WHO [7,8,9]; and Our World in Data [10], including the cumulative confirmed cases, deaths, and recovered cases per day. To better reveal the epidemic trends of COVID-19 in the two countries, the start time for data collection was the date of the first reported death (18 March 2020). This was the same date for both countries. The data collection timeframe ended at the closest feasible date to analysis (28 February 2022). Other initial values are outlined in Table S2 in the Supplementary Materials.

### 2.3. Data Analysis and Scenario Simulation

**Figure 1:**
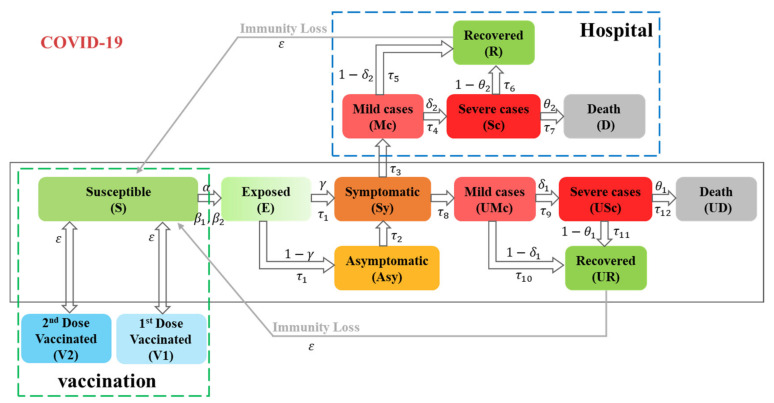
SEASIRD-HV model for COVID-19 transmission used for Pakistan and Bangladesh. Notes: Population was divided into susceptible (S), exposed (E), asymptomatic (Asy), symptomatic (Sy), infected (I), recovered (R) or dead (D), and vaccinated (V), where α is the contact rate, $\beta_{1},\;\beta_{2}$ are the infectivity of symptomatic cases and asymptomatic cases, γ is the proportion of exposed individuals transfer to symptomatic, and ε is the average immunological memory period. The infected (I) subjects are UMc, USc, Mc, and Sc, where $\delta_{1},\;\delta_{2}$ are the proportion of mild cases transforming to severe cases for the untreated and hospitalized conditions, respectively. The recovered (R) subjects are UR and R. The dead (D) subjects are UD and D, where $\theta_{1}\;\mathrm{and}\;\theta_{2}$ are the death ratios in untreated condition and hospital condition, respectively. $\tau_{1}$ to $\tau_{12}$ are the average time between current state and next state.

The model structure demonstrated in Figure 1 was mainly created to track the population N from susceptible (S) through exposed (E), asymptomatic (Asy), symptomatic (Sy), infected (I), and recovered (R) or dead (D) states, in cooperation with vaccination (V) states. The symptomatic (Sy) group is split among two pathways: one considers that these symptomatic cases are transferred to the hospital; the other considers that the rest of the symptomatic cases remain in the community without treatment. In particular, the infected population is divided into four compartments: the hospitalized mild cases (Mc), the hospitalized severe cases (Sc), the untreated mild cases (UMc), and the untreated severe cases (USc). The recovered population is divided into two compartments: the hospitalized recovered (R) and the untreated recovered (UR), and the same division applies to the dead population (hospitalized death (D) and untreated death (UD)). The vaccinated states include two stages: successfully vaccinated with the 1st dose of the vaccine (SV1), and successfully vaccinated with the 2nd dose of the vaccine (SV2). For this reason, the total population size (N) is assumed to be constant.

N = S + E + Sy + Asy + Mc + Sc + D + R + UMc +USc + UD + UR

Model formulations, assumptions, and parameter settings are provided in attached Supplementary Materials (pp. 2–4).

For data fitting, certain values were obtained from sources listed in Table S2 of Supplementary Materials. Remaining values were fit for the original data sources outlined above (data shown in the tab of “Reported data” in attached Supplementary Excel File for both countries). The calibration allows for less than 5% error. The calibrated model was then used to conduct counterfactual analysis to determine what could have happened if alternative measures were taken. Scenarios in this respect consider key changes to containment and control interventions including changing lockdown settings (timing, duration, and frequency), increasing hospital capacity, changing vaccination settings, and simultaneous changes of the above settings. Key outcomes of data fitting and counterfactual analysis are outlined in the following Section 3.

## 3. Results

### 3.1. Fitting Historic Data and Parameter Estimation

**Figure 2:**
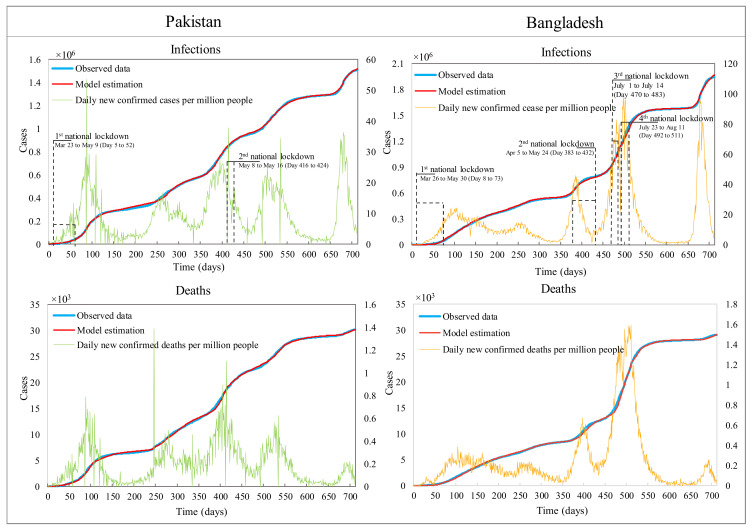
Simulation fitting curve results versus historic data. (**Left** axles) represents cumulative infections and deaths; (**right** axles) stand for daily cases of infections and deaths.

Since the outbreak of COVID-19 in Pakistan and Bangladesh, it appears that both countries experienced five waves of the COVID-19 pandemic within the chosen data timeframe. Pakistan imposed two national lockdowns and Bangladesh issued lockdowns four times, as shown in Figure 2. A piecewise calibration was carried out to estimate the necessary model parameters, given that the cumulative infections (CI) and cumulative deaths (CD) did not follow an expectedly exponential growth curve. This appeared due to the emergence of multiple COVID-19 variants [3] and multiple waves of transmission as well as the impact of various containment and control measures. Figure 2 shows that the simulation results were subsequently validated by achieving a very close fit with the historical data for both countries. 

### 3.2. Counterfactual Analysis

**Figure 3:**
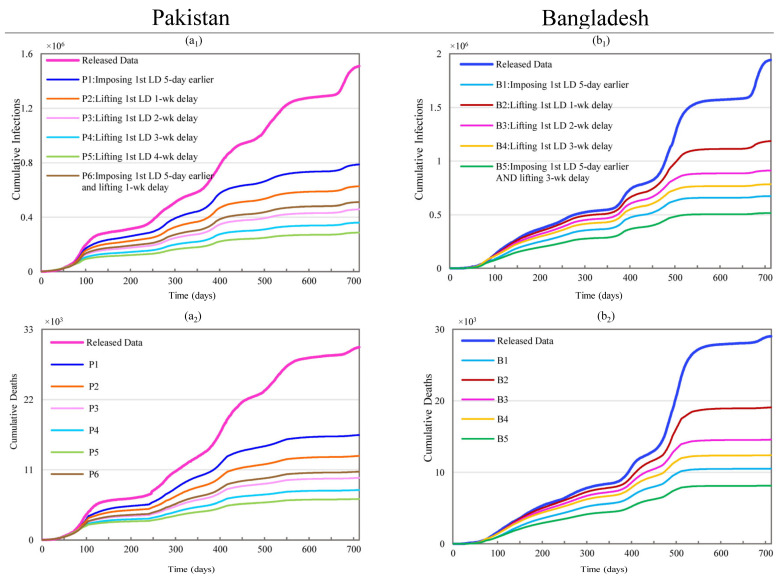
Effects of changing the timing and duration of the first lockdown on COVID-19 spread dynamics in Pakistan and Bangladesh. (**a_1_**,**a_2_**) represent simulation results of cumulative infections and deaths for scenarios P1 through P6 in Pakistan; (**b_1_**,**b_2_**) stand for simulation results of cumulative infections and deaths for scenarios B1 through B5 in Bangladesh.

**Figure 4:**
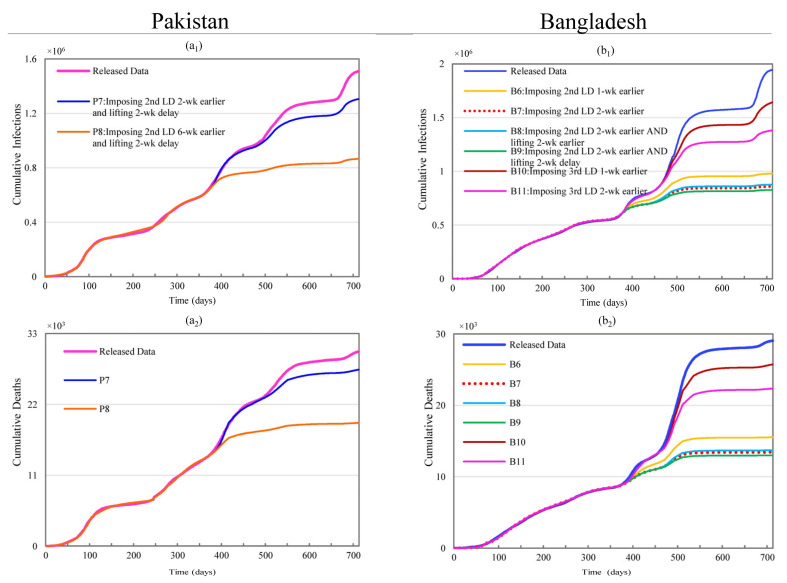
Impacts of changing timing for 2nd lockdown in Pakistan and 2nd, 3rd, and 4th lockdowns in Bangladesh. (**a_1_**,**a_2_**) represent simulation results of cumulative infections and deaths for scenarios P7 and P8 in Pakistan; (**b_1_**,**b_2_**) stand for simulation results of cumulative infections and deaths for scenarios B6 through B11 in Bangladesh.

**Figure 5:**
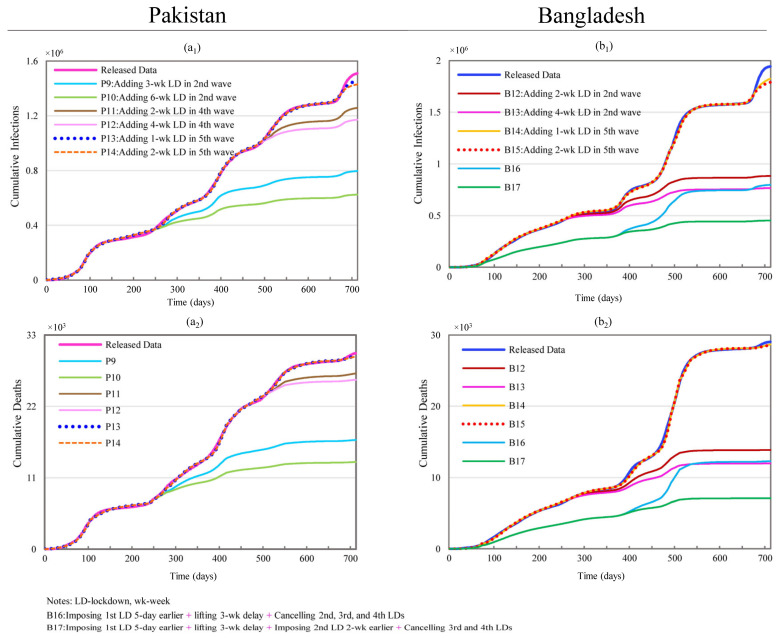
Effects of additional lockdowns on cumulative infections and cumulative deaths in Pakistan and Bangladesh. (**a_1_**,**a_2_**) represent simulation results of cumulative infections and deaths for scenarios P9 through P14 in Pakistan; (**b_1_**,**b_2_**) stand for simulation results of cumulative infections and deaths for scenarios B12 through B17 in Bangladesh.

**Figure 6:**
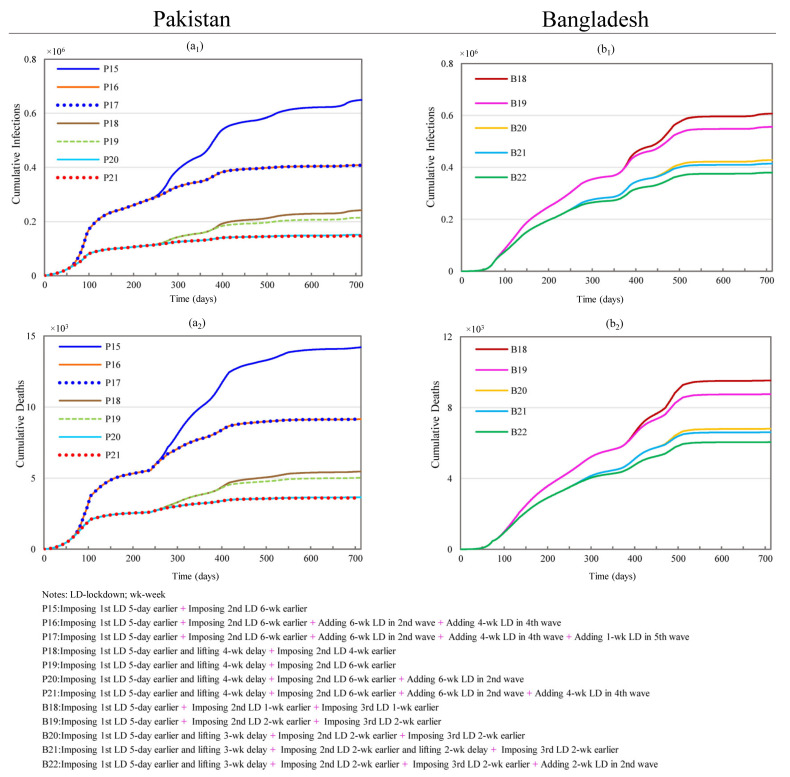
Effects of simultaneously changing multiple lockdowns on the cumulative infections and cumulative deaths in Pakistan and Bangladesh. (**a_1_**,**a_2_**) represent simulation results of cumulative infections and deaths for scenarios P15 through P21 in Pakistan; (**b_1_**,**b_2_**) stand for simulation results of cumulative infections and deaths for scenarios B18 through B22 in Bangladesh.

**Figure 7:**
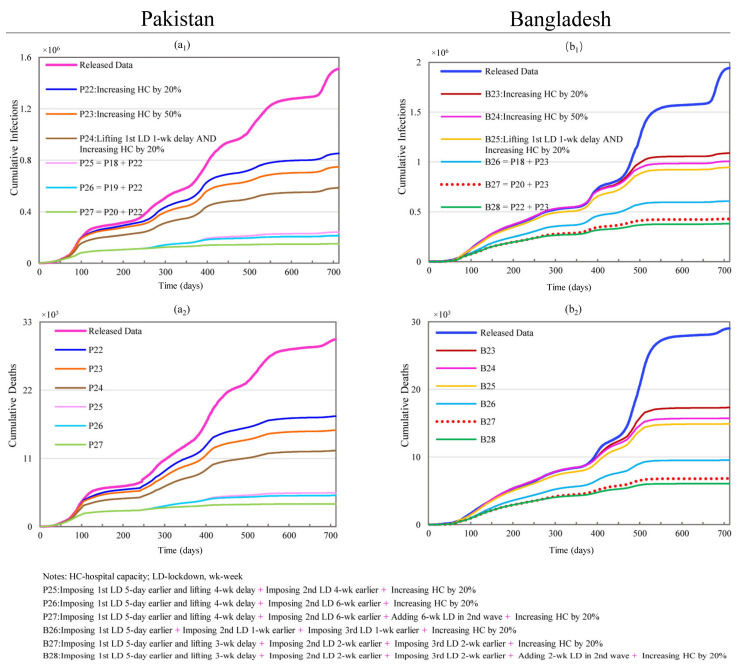
Effects of changing hospital capacity and resetting lockdowns on cumulative infections and cumulative deaths for Pakistan and Bangladesh. (**a_1_**,**a_2_**) represent simulation results of cumulative infections and deaths for scenarios P22 through P27 in Pakistan; (**b_1_**,**b_2_**) stand for simulation results of cumulative infections and deaths for scenarios B23 through B28 in Bangladesh.

**Figure 8:**
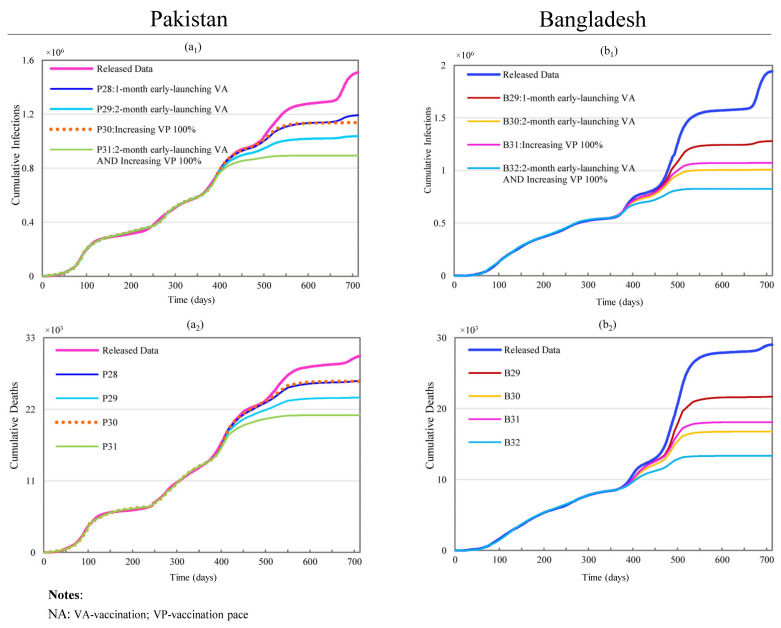
Effects of changing vaccination level on the cumulative infections and cumulative deaths for Pakistan and Bangladesh. (**a_1_**,**a_2_**) represent simulation results of cumulative infections and deaths for scenarios P28 through P31 in Pakistan; (**b_1_**,**b_2_**) stand for simulation results of cumulative infections and deaths for scenarios B29 through B32 in Bangladesh.

**Figure 9:**
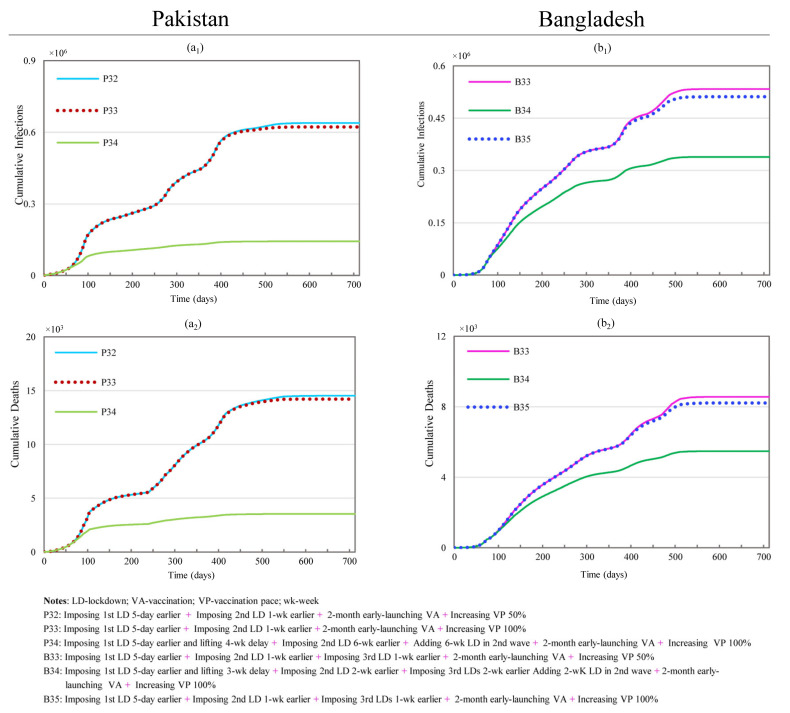
Effects of simultaneously changing lockdown, hospital capacity, and vaccination level on the cumulative infections and cumulative deaths for Pakistan and Bangladesh. (**a_1_**,**a_2_**) represent simulation results of cumulative infections and deaths for scenarios P32 through P34 in Pakistan; (**b_1_**,**b_2_**) stand for simulation results of cumulative infections and deaths for scenarios B33 through B35 in Bangladesh.

This section mainly conducts a counterfactual analysis to understand what could have happened with respect to the transmission dynamics by considering changes in the current timing windows and the durations of country-wide lockdowns, adding lockdowns, hospital capacities, and changing levels of vaccination. Combinations of each of these factors are also considered. For the sake of achieving a better visualization, except for those figures presented in the main text (i.e., Figures 3–9), the simulation results for further scenarios (Prefixed with PA and BA) are shown in the S4 Simulation Results section of the Supplementary Materials. In the same section, corresponding daily infections and deaths of simulated scenarios are also provided. The specific settings for the different scenarios are presented in the S3 Scenario Design section of the Supplementary Materials.

#### 3.2.1. Changing Initial Lockdown Timing and Duration

In Pakistan’s case, scenarios P1 through P6 investigate the effects of changing the lockdown timing and duration for the first countrywide lockdown imposed from 23 March to 9 May of 2020, as shown in Figure 3. Scenario P1, which assumes that the start of the first lockdown occurred 5 (The day reporting the first COVID-19 related death in Pakistan. Five days is also close to the incubation period of COVID-19. For ease of comparison, we also display the 5 day-shift in Bangladesh’s case.) days earlier than the original start date, sees a 47.83% decrease from 1.51 million to 0.79 million in CI and a 45.48% reduction in CD from 30,200 to 16,460. The simulation results of P2 to P5, which assumes a delay of 1, 2, 3, or 4 weeks for the lifting time of the first national lockdown, showed improved lockdown effectiveness while showing diminishing marginal returns as the duration of the lockdown is increased. Scenario P6, combining scenarios P1 and P2, resulted in reductions of 66.13% and 64.55% in CI and CD, which are close to the results of P3.

Scenarios B1 through B5 examine the effects of changing the timing and duration of Bangladesh’s first countrywide lockdown, which was imposed from 26 March to 30 May of 2020, as shown in Figure 3. Scenario B1 with the same assumption as P1 resulted in a 65.34% decrease in CI, from 1.94 million to 0.67 million infections, and a 63.76% reduction in CD, from 29,037 to 10,523 deaths. Scenarios B2 to B4 delay the lifting time of this lockdown by one, two, or three weeks. Scenario B3, which results in a 53.03% reduction in CI and a 49.77% reduction in CD, is the most notably favorable outcome when considering the impacts of the lockdowns and the diminishing marginal magnitudes with the increased duration of lockdown. A comparison between B1 and scenarios B2–B4 reveals that imposing the first lockdown 5 days earlier achieved an even better outcome than extending the lockdown by three weeks forward, as in scenario B4. Similar to the latter set of Pakistan scenarios, scenario B5 simulated the combinations of an earlier lockdown and extended lockdown durations. The effect of an earlier lockdown was so predominant that the combined scenarios could not generate any substantial reduction in CI or CD. Compared to B1, scenario B5 involved extending the lockdown by three weeks plus imposing the lockdown 5 days earlier, but it only resulted in additional reductions of 8.09% and 8.22%. 

#### 3.2.2. Changing Subsequent Lockdown Timing and Duration

Scenarios P7 and P8 in Figure 4 show the impacts of the second lockdown’s timing (also Figure S7 in the Supplementary Materials) on Pakistan. The second nationwide lockdown was imposed during the downslope period (about six weeks ahead of the wave trough-- please refer to the daily case curve in Figure 2) of the third wave. Scenario P7 generated non-significant reductions of 13.55% and 9.20% in CI and CD, where the lockdown was assumed to start two weeks earlier and last for an additional two weeks. Reductions in both CI and CD become much more pronounced when shifting the lockdown start times backward by more than three weeks. This could result in reductions of CI and CD as high as 42.65% and 36.63% in scenario P8, shown in Figure 4, where the lockdown was assumed to start six weeks earlier and last for an additional two weeks. 

Scenarios B6 through B9 in Figure 4 show the impacts of the second lockdown’s timing, while maintaining other conditions constant in Bangladesh. The results of scenarios B6 and B7 indicate that starting the lockdown one or two weeks early could result in considerable reductions in both CI (49.64% to 55.86%) and CD (46.51% to 53.70%). Scenarios B7 and B9 produced slightly larger reductions in CI (0.95% and 2.59%) and CD (0.92% and 2.47%) compared to scenario B8. However, scenario B8 did not increase the lockdown’s duration because the start and end dates were both moved backward. Nonetheless, this scenario achieved a 54.91% reduction in CI and a 52.78% reduction in CD.

Scenarios B10 and B11, shown in Figure 4, examine the impacts of starting the third national lockdown one and two weeks earlier, respectively. These impacts (CI: 15.56% and 28.99; CD: 11.33% and 23%) are remarkably smaller than the impacts of the equivalent scenarios for the first and second lockdowns. Increasing the duration of the third lockdown by delaying the end date did not substantially reduce CI and CD since it was very close to the third lockdown (only 9 days apart). Since the fourth lockdown was very close to the third lockdown (with only 9 days apart), increasing its duration by delaying the end date produced negligible reductions in CI and CD. 

#### 3.2.3. Changing Lockdown Frequency

This section, keeping other conditions constant, attempts to explore the impacts of changing the lockdown frequencies including adding lockdowns and canceling some current lockdowns in Pakistan and Bangladesh, as shown in Figure 5. Scenarios P9 and P10 add one lockdown with a respective duration of 3 and 6 weeks at the beginning of the upward slope of the second wave (starting from day 233) of the COVID-19 pandemic in Pakistan. The results show that P9 produced reductions of 58.69% and 55.51% in CI and CD and P10 led to reductions of 47.26% and 44.25%. 

Scenarios P11 and P12 show the impacts of adding two and four weeks of lockdown during the 4th wave (starting from day 482 onward). The maximum reductions in CI and CD are as high as 22.44% (338,920 cases) and 13.52% (4083 deaths) with a four-week lockdown. The reduction in the magnitudes of both the absolute values and percentages are a lot smaller than the impacts of an equivalent lockdown during the third wave, which are 51.81% (782,482 cases) and 48.80% (14,736 deaths) in CI and CD. Scenarios P13 and P14 examine the effects of adding one and two weeks of lockdown during the fifth wave (starting from day 666 onward). Compared to prior scenarios involving additional lockdowns, one additional lockdown during the fifth wave did not produce any substantial outcome. Two weeks of lockdown only resulted in a 5.31% reduction in CI and a 1.70% reduction in CD. 

Scenarios B12 to B15, shown in Figure 5, examine the effects of adding one countrywide lockdown in Bangladesh. Scenarios B12 and B13 show the impacts of adding one lockdown with a respective duration of two and four weeks at the beginning of the upward slope of the second wave of the COVID-19 pandemic (starting from day 242). The reductions in CI and CD could be as high as 60.61% (1,178,083) and 58.73% (17,052) with the duration of 4 weeks in B13. However, B12 achieved comparable reductions of 54.54% (1,060,055) and 52.21% (15,162) with only two weeks of lockdown. 

Scenarios B14 and B15 examine the impacts of one and two weeks of additional lockdown at the beginning of the upward slope of the fifth wave of the COVID-19 pandemic (Figure 5). A hypothetical two weeks of lockdown resulted in 7.89% (153,410) and 1.54% (446) reductions in CI and CD. This impact is much less than that of equivalent lockdowns imposed during the second wave of infection.

Scenario B16, simulating the cancelling of the second, third, and fourth countrywide lockdowns shown in scenario B5, can still produce reductions of 58.99% and 57.64% in CI and CD. Scenario B17 retains all the conditions from scenarios B5 and B7 while cancelling the third and fourth lockdowns. This generates reductions in CI and CD as high as 76.73% and 75.56%, which outperform the 73.43% and 71.98% reductions from scenario B8. 

#### 3.2.4. Effects of Changing Multiple Lockdown Conditions

Scenarios P15 to P17 combine various conditions from the scenarios outlined above and are shown in Figure 6. Scenario P15 involves starting the first lockdown and second lockdown 5 days and 6 weeks earlier, respectively. This scenario produces 9.16% and 7.45% more reductions in CI and CD than that of P1, which assumes the start of the first lockdown 5 days earlier. The maximum reductions in CI and CD are produced by scenario P17, with reductions of 72.99% and 69.70% in CI and CD, respectively. This scenario adds a further week to the fifth lockdown but only produces a negligible gain over the performance of scenario P16. The reductions produced under scenario P16 required about 15 additional weeks of lockdown. These impacts are nonetheless highly comparable to scenario PA1 (see Figures S2 and S23 in the Supplementary Materials), which only required 3 additional weeks of lockdown. 

Scenarios P18 through P21 are changed based on scenario PA3 (see Figures S2 and S23 in the Supplementary Materials), which produced substantial reductions in CI and CD (83.40% and 81.48%, respectively) by increasing the initial lockdown by five weeks (starting the 1st lockdown 5 days earlier and delaying the lifting date by 4 weeks). Scenario P21 results in substantial 90.24% and 88.04% reductions in CI and CD but these reductions require 16 additional weeks of lockdown, unlike scenario PA3. Likewise, P20 results in 6.57% and 6.41% more reductions in CI and CD compared to PA3 but requires 12 more weeks of lockdown. 

Scenario B18 is based on scenario B1 but entails starting both the second and third lockdowns 1-week earlier. B18 results in 3.41% and 3.40% more reductions in CI and CD compared to B1, shown in Figure 6. B19 involves starting the same lockdowns two weeks earlier and results in 6.03% and 6.06% more reductions than B1. Scenarios B20 to B22 were modified based on B5 (as they have the same conditions for the 1st lockdown) and attempt to investigate the joint effects of shifting the start and end dates for the lockdowns and adding additional lockdowns in Bangladesh. Scenario B20 with four more weeks of lockdown produces 4.54% and 4.56% more reductions in CI and CD compared to B5. Compared with B20, B21 has a two-week further duration of the second lockdown and results in less than 1% reductions in both CI and CD. Likewise, B22 also produces negligible improvements of the reductions in CI and CD compared to B5. The above results show that reductions in CI and CD are not very sensitive to additional lockdowns. This pales in comparison to the substantial benefits of the initial lockdown adjustments shown in Figure 4. 

#### 3.2.5. Hospital Capacity and Its Combination with Lockdowns

Scenarios P22 and P23 show that increasing hospital capacity can produce remarkable reductions in CI and CD, namely, 43.50% and 41.02% reductions with a 20% increase in hospital capacity, shown in Figure 7. Similar to the improvements due to lockdown timing and duration, hospital capacity also has a diminishing marginal effect on CI and CD, resulting in reductions of 50.36% and 48.50% with a 50% increase in capacity. In scenarios P24 through P27, an increased hospital capacity is combined with modified lockdown conditions. The simulations revealed that the increased hospital capacity in these combined scenarios has no additional benefit when the effects produced by changing the lockdown timing are remarkably large (P26, P27). Nonetheless, the increased hospital capacity produced additional benefits when combined with shorter lockdown durations, as demonstrated by comparing scenarios P2 with P24, where CI changes from a reduction of 58.45% to 61.16% and CD from a reduction of 56.37% to 59.36%. 

For Bangladesh, scenarios B23 and B24 show that an increased hospital capacity also results in considerable reductions in CI and CD (43.98% and 40.34% with a 20% increase in capacity), albeit with diminishing marginal effects (48.19% and 45.81% with a 50% increase in capacity), as shown in Figure 7. The marginal improvement between 20% and 50% increases in hospital capacity is slightly larger than in Pakistan. In scenarios B25 to B28, an increased hospital capacity is combined with lockdown timing changes, which were modified based on scenarios B2, B18, B20, and B22, respectively. Increased hospital capacity has no marginal benefit in scenarios B26 to B28 where the effects produced by changing the lockdown’s timing are remarkably large. Scenarios B18 (reductions of 68.76% and 67.16% in CI and CD), B20 (reductions of 77.97% and 76.54% in CI and CD), and B22 (reductions of 80.44% and 79.14% in CI and CD) are the corresponding scenarios without a hospital capacity increase, where the reductions generated by changing the lockdown’s timing are substantial. Nonetheless, an increased hospital capacity appears to offset the reduced impact of very short lockdown durations. This is demonstrated by comparing scenario B2 (reductions of 38.89% and 34.27% in CI and CD) with scenario B25 (reductions of 51.31% and 48.68% in CI and CD). 

#### 3.2.6. Changing Vaccination Setting and Lockdown Timing

Scenarios P28 and P29 show the impacts of the earlier administration of vaccinations in Pakistan. The results shown in Figure 8 reflect that the administration of vaccines one and two months earlier produces noticeable but marginally diminishing effects on CI and CD: 21.0% and 12.62% for P28; 31.30% and 21.12% for P29. Similar scenarios for Bangladesh led to larger impacts on CI and CD: 34.14% and 25.18% reductions in B29; 48.13% and 42.23% reductions in B30. 

Scenario P30 simulates a 100% increase in the vaccination capacity. It led to reductions of 24.70% and 12.85% in CI and CD. The same condition for Bangladesh resulted in reductions as high as 44.86% and 37.64% in scenario B31.

Scenario P31 simulates the joint impacts of combining early vaccination (P29) and increased vaccination capacity (P30) for Pakistan. The non-linear superposition of the individual effects resulted in an effect loss as high as 27.09% and 11.25% in the reductions of CI and CD compared to the direct sum of the effects of all the constituent scenarios (i.e., P29 + P30). For Bangladesh, under the same conditions, the effect losses were up to 38.11% in CI reduction and 32.33% in CD reductions, as shown in Figure 8.

Scenarios P32 through P34 examine the joint effects of modifying both the vaccination settings and lockdown timings, as shown in Figure 9. Although the maximum effect is obtained by P34 (with reductions of 90.54% and 88.26% in CI and CD), the strongest combined effect was produced by P33, which showed marginal increases of 10.55% and 7.15%, compared with only 0.57% and 0.38% for P34. When considering the availability of vaccines, P32 (with reductions of 57.66% and 51.89% in CI and CD) may be the best option since the marginal increases in the reductions of CI and CD were 9.44% and 6.14%. These impacts only require a 50% increase in vaccine supply, instead of the 100% increase for scenario P33.

Scenarios B33–B35 simulate the impacts of changing both vaccination settings and lockdown timings for Bangladesh, as shown in Figure 9. As mentioned previously, with the same level of changes, the effects generated in Bangladesh are more sensitive than those in Pakistan. Under the conditions of changing the lockdown’s timing, the reductions in both CI and CD exceed 67%. This shows that marginal contributions from simultaneous vaccination adjustments are very small, with a maximum additional impact of less than 5%. Within this group of scenarios, B33 and B35 have the shortest lockdown durations while having noticeably marginal effects (more than 3%) with modified vaccination conditions. Scenario B33 (with reductions of 72.54% and 70.50% in CI and CD) is preferable to scenario B35 (with reductions of 73.69% and 71.71% in CI and CD) because B33 requires the vaccination speed to increase by 50% rather than the 100% for B35. 

## 4. Discussion

The key outcome measures were clearly impacted by the lockdown’s timing and duration in a range of the simulated scenarios. The simulations also indicated distinct trajectories for Bangladesh and Pakistan, despite the many characteristics shared by these countries. Similarities and differences were also discussed for hospital capacity adjustments, and for vaccination-related adjustments. The combination of changes in lockdown timing, duration, and frequency under-performed compared with the sum of the discrete contributions for the non-combined interventions. After the discussion of these factors, the comparative strengths and limitations of the current analyses are outlined; then, the implications for pandemic management policies and further research are presented. 

### 4.1. Lockdown Adjustments

Some of the results concerning the initial lockdowns varied between the two countries. Nonetheless, earlier lockdowns improved the intervention outcomes in simulations for both national settings. Increased lockdown durations also increased the CI and CD reductions in both Bangladesh and Pakistan. While an earlier initial lockdown would have been particularly effective in Bangladesh, increasing the duration of the lockdown by delaying the lifting date would have been particularly beneficial for Pakistan. The diminishing marginal benefits over time of the lockdown in both countries highlight the need for balancing the duration of lockdowns, its impacts on infection trajectories, and the negative impacts of lockdown interventions [44,45,46]. 

Simulating a two-week extension of lockdowns in Pakistan resulted in a 69.65% reduction in CI and a 67.79% reduction in CD. This lockdown timeframe was also applied to the fourth wave of infections in Pakistan. A two-week (scenario P11) lockdown appeared to be the optimal response to this phase, considering the minimal impact of longer durations. Comparable results were also achieved for Bangladesh by adjusting the timing of the second lockdown. The results of the simulation scenario B8 suggest that Bangladesh could have achieved better containment and control with an earlier second lockdown, without a clear need to extend the duration of the lockdown. Moreover, regarding the effects of changing the second lockdown, significant containment outcomes could be achieved in Pakistan by starting their lockdown six weeks earlier; in Bangladesh, this could be achieved by shifting the start date of the second lockdown one week backward. This indicates that Bangladesh’s choice of timing window in the second lockdown is closer to an ideal situation than that of Pakistan, which also reveals that containment outcomes are more time-sensitive to the duration of the lockdown.

An additional lockdown appeared to be effective in the context of Bangladesh, in addition to the four lockdowns originally imposed. The simulated reductions in CI and CD were as high as 60.61% (1,178,083) and 58.73% (17,052), with a duration of 4 weeks. However, scenario B12 achieved comparable reductions of 54.54% (1,060,055) and 52.21% (15,162) with only two weeks of lockdown. Once again, a two-week duration appeared to achieve the gross majority of lockdown benefits. 

Some of the lockdowns were more effective than others in a range of the simulated scenarios. For example, scenario B17 involved cancelling the 3rd and 4th lockdowns in combination with the conditions from scenarios B7 and B5 (i.e., extending the second lockdown by three weeks and starting it two weeks earlier). The resulting scenario sustained a 76.73% reduction in CI and a 75.56% reduction in CD, outperforming the 73.43 and 71.98% reductions by retaining the original set of lockdowns. This is another example of the importance of lockdown timing, and particularly the effectiveness of early lockdowns.

### 4.2. Hospital Capacity and Vaccination

An increased hospital capacity appeared to offset the small impacts of the minimal adjustments to the duration of the lockdown. This is demonstrated by comparing scenario B2, adding only two weeks to the initial lockdown, to Scenario B25, involving a minimal increase in hospital capacity. The shorter lockdown achieved a 38.89% reduction in CI and a 34.27% reduction in CD. Combined with improved hospital capacities, this improved to a 51.31% reduction in CI and a 48.68% reduction in CD. The equivalent simulations for Pakistan showed that differences in hospital capacities would have had an even greater positive impact on reducing the infection outcomes, which is demonstrated by the pair of P2 vs. P24. 

The reverse appears to apply to vaccination-related adjustments. Comparing scenario P30 with the Bangladesh equivalent (B31) suggests that COVID-19 vaccination capacities led to more substantial impacts for Bangladesh. Both scenarios involved doubling vaccination capacities, but the Bangladesh scenario resulted in 44.86% and 37.64% reductions in CI and CD. These reductions only reached 24.70% and 12.85% for the equivalent scenario in Pakistan. Bangladesh’s reduction in CI and CD is more sensitive to both an early launch of vaccination and an increase in vaccination capacity than Pakistan, where a doubling vaccination capacity matters more. A doubling vaccination capacity in Bangladesh produces twice as much of a reduction in CI as Pakistan and three times the reduction in CD as Pakistan.

### 4.3. Combined Adjustments

In the cases of simultaneous adjustments to multiple lockdowns, the simulation results for both countries have revealed that changes in subsequent lockdowns can only generate trivially marginal effects if the changes in the preceding lockdowns can produce remarkable effects. This is also observed in the scenarios for combined adjustments to hospital capacity and vaccination (launching date and administration capacity). Under the circumstance of an increasing vaccine supply and administration capacity, combining lockdown and vaccination could generate significant effects on the reductions of CI and CD. With the same level of changes in the combination mentioned above, the effects in Bangladesh were much larger than those of Pakistan. The simulation results showed that in both countries, the marginal effects of increasing vaccination capacity became noticeable when the lockdown durations were reduced. In the case of vaccine undersupply, the scenarios combining a shortened lockdown duration and vaccination can still produce remarkable effects towards reducing the CI and CD. Under same conditions, the effects generated in Bangladesh were more significant than those in Pakistan, which means Bangladesh was more responsive to the proposed interventions. 

Therefore, to reduce unnecessary negative externalities to socioeconomic activities, policy/decision makers could discretionally take the situation of the “counterproductive effect” into consideration when determining the combined containment measures. Regarding the effects of adopting different containment and control strategies or their combinations, what policy makers or decision makers should pursue are not the maximum values under extreme conditions, such as very long lockdown times, but a balanced compound strategies where joint effects are synergistically augmented and collateral damages are minimized while still achieving remarkable containment outcomes, such as effectively flattening the pandemic curve and reducing the CI and CD. The planning of the timing and duration of lockdowns should be contingent on the availability of alternative containment measures and resources such as hospital capacity and the level of vaccination to reduce overresponses to the outbreak. The design and implementation of containment and control strategies must also be tailored to specific contexts formed by socioeconomic environments and public health governance. 

### 4.4. Strengths and Limitations

The key strength of the current research was the assessment of scenarios including both pharmaceutical and non-pharmaceutical interventions. These interventions were incorporated into a simulation framework to understand the differences in their dynamic impacts on the spread of COVID-19 in two countries sharing some similar socio-demographic characteristics; in this case, two countries that were administered as a single state for 24 years under the “One Unit Scheme” program but have exercised different public health governance practices. The second key strength of this study was the ability to capture the effects of both normal social activities and major social events (such as religious events and national holidays) by incorporating piecewise social contact characteristics and corresponding containment measures. The current research also demonstrated that the SDM was able to simultaneously account for different COVID-19 spread trajectories in a counterfactual analysis under a wide range of interventions and their combinations. Therefore, this study can provide a roadmap and framework to help gain a better understanding of the issues and to develop ways in which to design and implement pertinent interventions under different assumptions, such as stringencies in controlling social activities, the enforcement of social distancing, vaccination capacity, hospital capacity, and the new characteristics of SARS-CoV-2 variants. The current SDM can be easily modified to understand the spread dynamics and impacts of the corresponding interventions of emerging infectious diseases.

As in all research, the current analysis has certain limitations. First, all the data were aggregated at the national level without capturing regional heterogeneity. Although the current analysis addressed a range of interventions, this range was not exhaustive and was subject to the public availability of the relevant data. Data regarding the economic impacts and intervention costs were not available at the time of analysis; thus, the current research does not constitute a comprehensive policy analysis. Likewise, the continuing mutations of the COVID-19 virus mean that the current analysis may not reflect future dynamics within the current pandemic. 

### 4.5. Practical Implications and Future Directions

Our study provides a way for researchers, policy/decision makers, and practitioners to gain a better understanding of the degree of difference the same (or similar) interventions can produce in two countries, where the modeling mechanisms and results can facilitate international comparisons with contributions to enhance global public health governance. The current results indicate how optimal lockdown windows can be identified for both Bangladesh and Pakistan. The results demonstrate that alternative lockdown strategies are particularly effective but are also subject to the dynamic availability of resources such as hospital capacity and vaccine supplies. Effective strategies can reduce negative socioeconomic impacts by limiting the disruption caused by extended lockdowns and other extreme measures while still achieving containment and control. The results can inform decision makers of the smart combination of multiple interventions to avoid wasting resources since the effects produced by some interventions diminish with the increase in the relevant resources or the level of stringencies. 

The current SDM has been able to incorporate factors of supply and demand for resources such as face masks, vaccines, and medical facilities. It has also been able to account for the availability and allocation of fiscal resources. Future research can use this approach to investigate how these resources, and the required funding, affect control and containment outcomes. Although each pandemic is distinct, it is anticipated that the current model can be extended to improve the design, planning, and implementation of interventions/policies for future emerging diseases. 

## 5. Conclusions

The COVID-19 pandemic has highlighted the importance of the control and containment interventions and the importance of combining these interventions in an optimal manner during progressive phases and waves of the pandemic. The ensuing outcomes, concerning a range of countries with different sociodemographic characteristics and public health governance structures, have triggered heated debates in the academic literature and the public health policy arena. However, cross-country comparisons on the relative impacts of common interventions remain scarce. This situation demands in-depth analyses of how interventions perform in different social contexts, to enhance mutual understanding and cooperation between different countries, thereby bringing those countries together to consolidate more global responses to a global health crisis and to provide enduring improvements to wider global health governance. 

The SEASIRD-HV model developed in this study attempts to capture COVID-19’s transmission dynamics and the responsiveness of Pakistan and Bangladesh to the proposed interventions, i.e., adjusting country-wide lockdowns, changing hospital capacities, and resetting vaccination timing and capacity. The results support the notion that country-wide lockdowns have played an extremely important role in controlling the spread of COVID-19 and in flattening the curve in countries with low vaccination levels. An early lockdown would be especially effective. Our simulation results also show that scenarios combining an increased hospital capacity and a shortened lockdown can produce prominent effects towards reducing CI and CD. With the same assumptions for lockdowns, increasing the hospital capacities in Pakistan could have had a larger impact than that of Bangladesh. Moreover, our findings reveal that Bangladesh was more sensitive to both early-launched vaccinations and changes in the vaccination levels. To reduce unnecessary negative externalities caused by certain containment measures, such as long lockdown durations, our simulation results can help find balanced points to leverage the impacts of the combined interventions. 

The current paper has provided a systematic evaluation of the impacts of relevant interventions and their combinations on the COVID-19 containment outcomes in Bangladesh and Pakistan by considering the stringencies of containment strategies, and the logistical challenges of distributing resources such as vaccines and hospital capacity. The current research provides a workable model for relevant analyses, and several practical conclusions regarding lockdown timing. An even more complete and useful understanding of the relative benefits could be generated by comparable research in a wider range of social contexts, and this presents an aspirational challenge for scholars around the world. 

## Data Availability

Where possible, data and mathematical models have either been provided in full, or they have been linked to the manuscript or Supplementary Materials.

## Supplementary Materials

The following supporting information can be downloaded at: https://www.mdpi.com/article/10.3390/ijerph19159239/s1. A substantial volume of Supplementary Materials is included for online publication [47,48,49,50,51,52,53,54,55,56,57,58,59,60,61,62,63,64,65,66,67,68,69,70,71,72,73,74,75,76,77,78,79,80,81,82,83,84,85,86,87,88,89,90,91,92,93,94,95,96,97,98,99,100,101,102,103,104,105,106,107,108,109,110,111,112].
