# Supplementary material for: Assessing the Dynamic Outcomes of Containment Strategies against COVID-19 under Different Public Health Governance Structures: A Comparison between Pakistan and Bangladesh
Source: Int J Environ Res Public Health. 2022 Jul 28;19(15):9239. doi: 10.3390/ijerph19159239 (PMC9368361; doi:10.3390/ijerph19159239)
Supplement: Supplementary file 1 [file ijerph-19-09239-s001.zip › ijerph-1792872-supplementary.pdf]

Supplementary Materials

S1 COVID-19 Outbreak Situations in Pakistan and Bangladesh

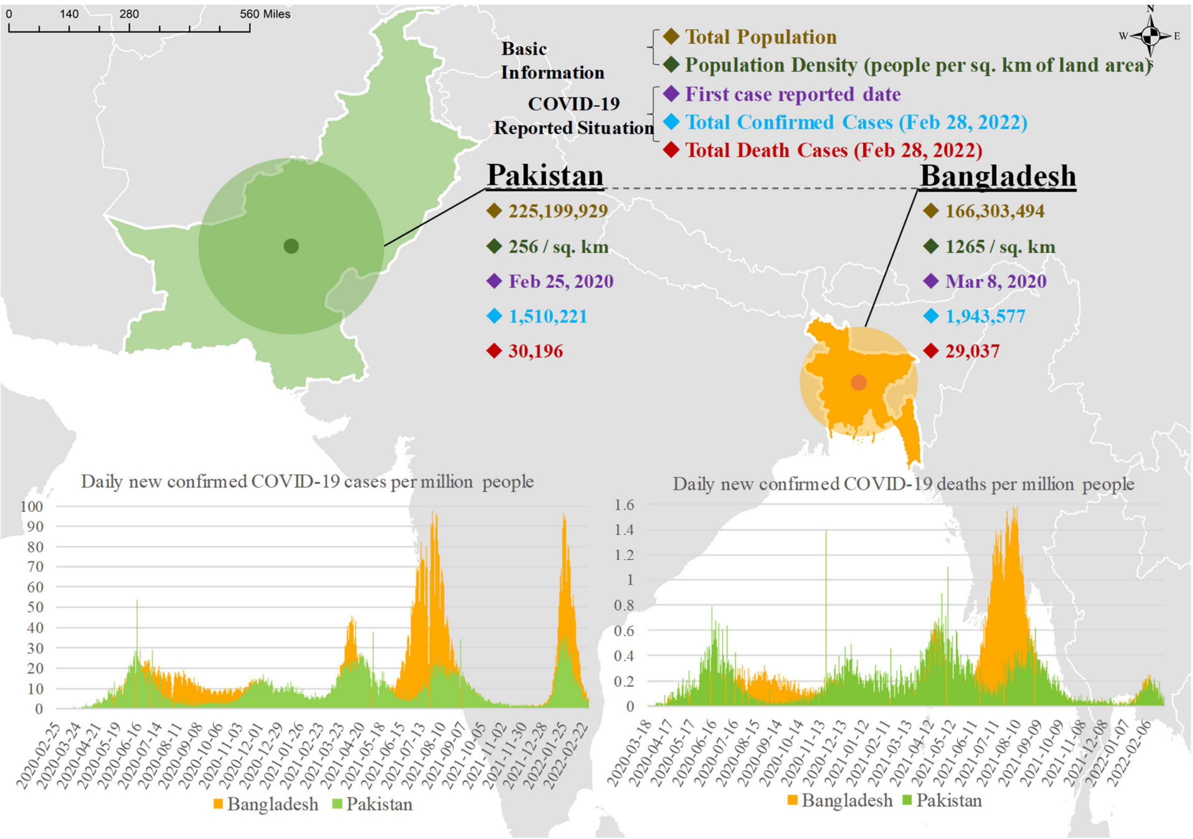

Source: Our World in Data and Johns Hopkins University Center for Systems Science and Engineering (CSSE) COVID-19 Data. The comparing daily new confirmed cases data with Pakistan and Bangladesh was relative to the size of the per million people.

Figure S1. The portrayal of COVID-19 outbreak situations in Pakistan and Bangladesh

S2 Model Formulations, Assumptions, and Parameter Settings

Dynamics of these compartments across time were described by the following set of ordinary differential equations. Notations used in these core equations are defined in Table S1. At a specific time t, the number of individuals in the initial stage and subsequent conditions are derived by:

$$\frac{dS(t)}{dt} = \frac{R(t)+UR(t)+SV1(t)+SV2(t)}{\varepsilon} - \lambda_1 * \min\left(\min\left(\mu_1, \frac{TV1(t)}{\omega}\right), \frac{S(t)}{\omega}\right) - \min\left(\frac{\sigma*(\lambda_2-\lambda_1)}{\lambda_1}, \min\left(\min(\mu_2, \frac{TV2(t)}{\omega}), \frac{S(t)}{\omega}\right)\right) - \frac{S(t)*\alpha*\beta_1*(SY(t)+UMc(t)+USc(t))}{N} - \frac{S(t)*\alpha*\beta_2*AY(t)}{N} \quad (1)$$

$$\frac{dE(t)}{dt} = \frac{S(t)*\alpha*\beta_1*(SY(t)+UMc(t)+USc(t))}{N} + \frac{S(t)*\alpha*\beta_2*AYC(t)}{N} - \frac{E(t)*\gamma}{\tau_1} - \frac{E(t)*(1-\gamma)}{\tau_1} \quad (2)$$

$$\frac{dSY(t)}{dt} = \frac{E(t)*\gamma}{\tau_1} + \frac{AY(t)}{\tau_2} - \frac{\min(SY(t), H-Mc(t)-Sc(t))}{\tau_3} - \frac{SY(t)}{\tau_8} \quad (3)$$

$$\frac{dAY(t)}{dt} = \frac{E(t)*(1-\gamma)}{\tau_1} - \frac{AY(t)}{\tau_2} \quad (4)$$

$$\frac{dMc(t)}{dt} = \frac{\min(SY(t), H-Mc(t)-Sc(t))}{\tau_3} - \frac{Mc(t)*(1-\delta_2)}{\tau_5} - \frac{Mc(t)*\delta_2}{\tau_4} \quad (5)$$

$$\frac{dSc(t)}{dt} = \frac{Mc(t)*\delta_2}{\tau_4} - \frac{Sc(t)*(1-\theta_2)}{\tau_6} - \frac{Sc(t)*\theta_2}{\tau_7} \quad (6)$$

$$\frac{dR(t)}{dt} = \frac{Sc(t)*(1-\theta_2)}{\tau_6} + \frac{Mc(t)*(1-\delta_2)}{\tau_5} - \frac{R(t)}{\varepsilon} \quad (7)$$

$$\frac{dD(t)}{dt} = \frac{Sc(t)*\theta_2}{\tau_7} \quad (8)$$

Untreated condition

$$\frac{dUMc(t)}{dt} = \frac{SY(t)}{\tau_8} - \frac{UMc(t)*(1-\delta_1)}{\tau_{10}} - \frac{UMc(t)*\delta_1}{\tau_9} \quad (9)$$

$$\frac{dUSc(t)}{dt} = \frac{UMc(t)*\delta_1}{\tau_9} - \frac{USc(t)*(1-\theta_1)}{\tau_{11}} - \frac{USc(t)*\theta_1}{\tau_{12}} \quad (10)$$

$$\frac{dUR(t)}{dt} = \frac{USc(t)*(1-\theta_1)}{\tau_{11}} + \frac{UMc(t)*(1-\delta_1)}{\tau_{10}} - \frac{UR(t)}{\varepsilon} \quad (11)$$

$$\frac{dUD(t)}{dt} = \frac{USc(t)*\theta_1}{\tau_{12}} \quad (12)$$

Vaccination

$$\frac{dTV1(t)}{dt} = \nu_1 - \min(\mu_1, \frac{TV1(t)}{\omega}) \quad (13)$$

$$\frac{dTV2(t)}{dt} = \nu_2 - \min(\mu_2, \frac{TV2(t)}{\omega}) \quad (14)$$

$$\frac{dSV1(t)}{dt} = \lambda_1 * \min\left(\min\left(\mu_1, \frac{TV1(t)}{\omega}\right), \frac{S(t)}{\omega}\right) - \frac{SV1(t)}{\varepsilon} \quad (15)$$

$$\frac{dSV2(t)}{dt} = \min\left(\frac{\text{DELAY FIXED}(SV1(t),\sigma,0)*(\lambda_2-\lambda_1)}{\lambda_1}, \min\left(\min(\mu_2, \frac{TV2(t)}{\omega}), \frac{S(t)}{\omega}\right)\right) - \frac{SV2(t)}{\varepsilon} \quad (16)$$

Table S1. Notation description for variables and parameters used in the model

Notation	Description
S(t), E(t), Sy(t), Asy(t)	The states of disease transmission at initial stage: Susceptible, Exposed, Symptomatic, Asymptomatic
UMc(t), USc(t), UD(t), UR(t)	The states of disease transmission in untreated condition: Untreated Mild cases, Untreated Severe cases, Untreated Death, and Untreated Recovered
Mc(t), Sc(t), D(t), R(t)	The states of disease transmission in hospital: Mild cases, Severe cases, Death, and Recovered
SV1(t), SV2(t)	The population protected by successfully vaccinating: 1st dose; 2nd dose
TV1(t), TV2(t)	Total available vaccine capacity: 1st dose; 2nd dose
N	Total population
H	Total COVID-19 dedicated hospital beds
α	Contact rate
β_1, β_2	The Infectivity of Symptomatic cases, Asymptomatic cases
γ	The proportion from Exposed to Symptomatic
δ_1, δ_2	The proportion from Mild cases to Severe cases: in untreated condition, in hospitalization condition
θ_1, θ_2	The death rate: in untreated condition, in hospitalization condition
τ_1, τ_2, τ_3	The incubation period; time from Asymptomatic to Symptomatic; diagnosis time (also is the time from Symptomatic to Mild in hospitalization condition)
$\tau_4, \tau_5, \tau_6, \tau_7$	The time between the disease spread states in hospitalization condition: Mild – Severe; Mild – Recovered; Severe – Recovered; Severe – Death
$\tau_8, \tau_9, \tau_{10}, \tau_{11}, \tau_{12}$	The time between the disease spread states in untreated condition: Symptomatic – Mild; Mild – Severe; Mild – Recovered; Severe – Recovered; Severe – Death
ν_1, ν_2	The vaccine supply capacity: 1st dose; 2nd dose
ε	The average immunological memory period
μ_1, μ_2	The actual administering capacity: 1st dose; 2nd dose
λ_1, λ_2	The effectiveness: 1st dose; 2nd dose
σ	The interval for administering 1st and 2nd dose vaccines
ω	The transition time length unit (1 day)

The following assumptions were also made, while deriving the initial model shown in Figure 2 in the manuscript:

1) Susceptible (S) individuals are exposed or infected through contact with infectious individuals, including asymptomatic cases or symptomatic cases.
2) With an average incubation period of 5.8 days [40, 47], exposed individuals either transfer to symptomatic (Sy) or asymptomatic (Asy), not all infected individuals exhibit symptoms.
3) The virus-infected individuals are not infectious during the incubation period.
4) The proportion from exposed to symptomatic is 0.85 [48, 49].
5) The available hospital capacity is limited, only parts of symptomatic cases can be transferred to hospital, and the rest cases within untreated condition.
6) Symptomatic cases were quarantined in the hospital.
7) Individuals with mild infection either recovered (R or UR) or worsen to a severe case (Sc or USc).
8) Individuals with severe infection either recovered (R or UR) or worsen to death (D or UD).
9) Recovered (R or UR) individuals will return to the susceptible (S) group with a temporary immunity of 240 days.
10) The effectiveness of 1st dose and 2nd dose vaccination is 0.6 [50,51], 0.8 [50,52], respectively.
11) Vaccinated Individuals have a temporary immunity of 240 days [53-55].

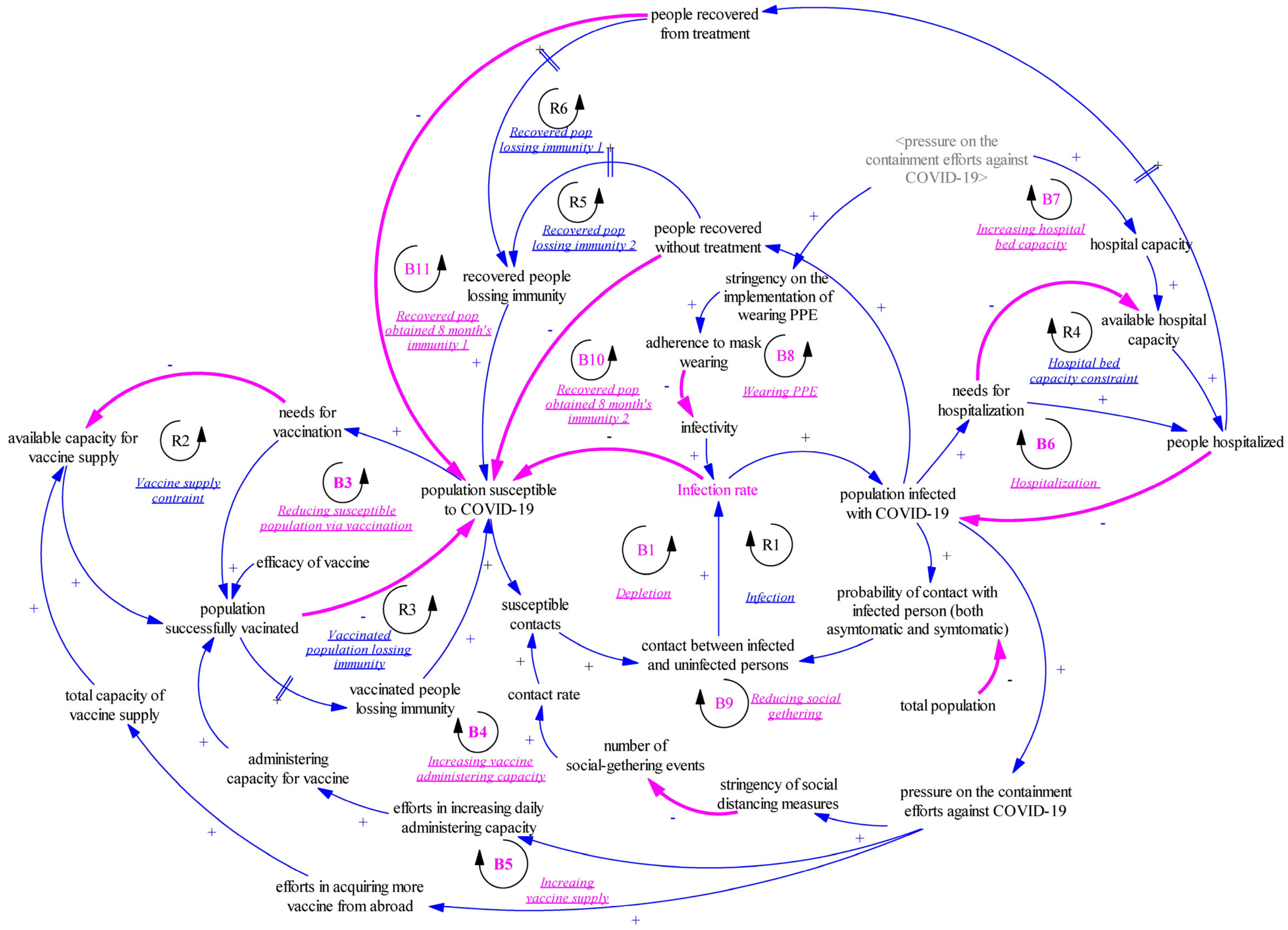

Figure S2. Causal loop diagram for displaying feedback loops in the epidemiological system dynamics model

Table S2. Parameter values used in the model

Parameters	Description	Bangladesh		Pakistan	
		**Initial values**	**Sources**	**Initial values**	**Sources**
S(0)	Susceptible population	164,689,211	Calculated	220887290	Calculated
E(0)	Exposed population	150	Assumed	3050	Assumed
Sy(0)	Symptomatic cases	3	Assumed	50	Assumed
Asy(0)	Asymptomatic cases	1	Assumed	25	Assumed
UMc(0)	Untreated confirmed mild cases	0	Assumed	0	Assumed
USc(0)	Untreated severe cases	0	Assumed	0	Assumed
UD(0)	Untreated death population	0	Assumed	0	Assumed
UR(0)	Untreated recovered population	0	Assumed	0	Assumed
Mc(0)	Hospitalized mild cases	11	Assumed	364	Assumed
Sc(0)	Hospitalized severe cases	3	Assumed	90	Assumed
D(0)	Hospitalized death population	1	Empirical data [10]	2	Empirical data [10]
R(0)	Hospitalized recovered population	3	Empirical data [56]	13	Empirical data [57]
SV1(0)	The population was successfully vaccinated 1st dose	0	Empirical data [58]	0	Empirical data [10]
SV2(0)	The population was successfully vaccinated 2nd dose	0	Empirical data [58]	0	Empirical data [10]
TV1(0)	Total available 1st dose vaccine capacity	0	Empirical data [58]	0	Empirical data [10]
TV2(0)	Total available 2nd dose vaccine capacity	0	Empirical data [58]	0	Empirical data [10]
N	Total population	164,689,383	Empirical data [59]	220,892,331	Empirical data [60]
H	Total COVID-19 dedicated hospital beds	20094	Average from [61]	22001	Average from [62]
α	Contact rate	Table S3	Calibrated	Table S6	Calibrated
β_1	Infectivity of Symptomatic	Table S4	Calibrated and referred to [63,64]	Table S7	Calibrated and referred to [63,64]
β_2	Infectivity of Asymptomatic cases	Table S4	Calibrated and referred to [63]	Table S7	Calibrated and referred to [63]
γ	Proportion from Exposed to Symptomatic	0.85	[48,49]	0.85	[48,49]
δ_1	Proportion from Mild cases to Severe cases in untreated condition	0.0200198	Calibrated	0.0299991	Calibrated
δ_2	Proportion from Mild cases to Severe cases in hospitalization condition	0.0110016	Calibrated and referred to [41,65]	0.0118299	Calibrated and referred to [41,65]
θ_1	Death rate in untreated condition	Table S5	Calibrated and referred to [66]	Table S8	Calibrated and referred to [66]

θ_2	Death rate in hospitalization condition	Table S5	Calibrated and referred to [67]	Table S8	Calibrated and referred to [67]
τ_1	Incubation period	5.8	[40,50]	5.8	[40,50]
τ_2	Time from Asymptomatic to Symptomatic	5.85347	Calibrated and referred to [68]	7.01039	Calibrated and referred to [68]
τ_3	Diagnosis time (Time from Symptomatic to Mild in hospitalization condition)	0.717016	Calibrated and referred to [69,70]	0.500968	Calibrated and referred to [69,70]
τ_4	Time from mild to severe in hospitalization condition	5.99938	Calibrated and referred to [63, 71]	4.36935	Calibrated and referred to [63, 71]
τ_5	Time from mild to recovered in hospitalization condition	9.82998	Calibrated and referred to [72]	10.0593	Calibrated and referred to [72]
τ_6	Time from severe to recovered in hospitalization condition	15.4493	Calibrated and referred to [73]	17.0006	Calibrated and referred to [73]
τ_7	Time from severe to death in hospitalization condition	3.17026	Calibrated and referred to [63]	4.02109	Calibrated and referred to [63]
τ_8	Time from symptomatic to mild in untreated condition	0.967464	Calibrated	0.75036	Calibrated
τ_9	Time from mild to severe in untreated condition	3.00679	Calibrated	3.14916	Calibrated
τ_{10}	Time from mild to recovered in untreated condition	12.4416	Calibrated	12.894	Calibrated
τ_{11}	Time from severe to recovered in untreated condition	21.0524	Calibrated	21.5682	Calibrated
τ_{12}	Time from severe to death in untreated condition	1.18197	Calibrated	2.99997	Calibrated
ν_1	1st dose vaccine supply capacity	Empirical data	[58]	Empirical data	[10]
ν_2	2nd dose vaccine supply capacity	Empirical data	[58]	Empirical data	[10]
ε	Average immunological memory period	240	[53-55]	240	[53-55]
μ_1	1st dose administering capacity	Empirical data	[58]	Empirical data	[10]
μ_2	2nd dose administering capacity	Empirical data	[58]	Empirical data	[10]
λ_1	1st dose vaccine effectiveness	60%	[50,51]	70%	[50,51]
λ_2	2nd dose vaccine effectiveness	80%	[50,52]	90%	[50,52]
σ	Delay time for administering the 2nd dose vaccine	14 days	[51,52]	14 days	[51,52]
ω	Time length unit	1	Assumed	1	Assumed

Table S3. Calibrated average contact rate at different period in **Pakistan**

Actual date	Model time	Contact Rate	Calibration Value	Policy and social activity	Sources
2020/03/18-03/23	0-5	Contact Rate1	16.2189	School closure but No lockdown	[74]
2020/03/23-05/09	5-52	Contact Rate2	9.4982	1st countrywide lockdown	[75]
2020/05/09-06/16	52-90	Contact Rate3	16.5746	Ramadan (Apr 25 to May 25, 2020)	[76]
2020/06/16-06/30	90-104	Contact Rate4	11.0028	Partial city lockdown (moderate control gradually strict)	[77]
2020/06/30-08/29	104-164	Contact Rate5	16.9716		
2020/08/29-11/13	164-240	Contact Rate6	12.5819	Ashuara (Aug 29 to 30, 2020)<br>Eid Milad un Nabi (Oct 29 to 30, 2020)	[78,79]
2020/11/13-12/21	240-278	Contact Rate7	13.1216	Ijtima (Nov 15 to 20, 2020)	[80]
202012/21-2021/03/01	278-348	Contact Rate8	11.0007	Quaid-e-Azam Day (Dec 25, 2021)	[81]
2021/03/01-04/11	348-389	Contact Rate9	15.7805		
2021/04/11-05/08	389-416	Contact Rate10	10.8112	Ramadan (Apr 13 to May 13, 2021)	[82]
2021/05/08-05/16	416-424	Contact Rate11	9.4925	2nd countrywide lockdown	[83]
2021/05/16-07/01	424-470	Contact Rate12	11.1818	Partial city lockdown or smart lockdown (strict control)	[84]
2021/07/01-08/18	470-518	Contact Rate13	15.7386	Partial city lockdown or smart lockdown (strict control)<br>Eid ul Adha (Jul 20 to 22, 2021)<br>Muharram (Aug 9 to 18, 2021)<br>Independence Day (Aug 14, 2021)	[84-88]
2021/08/18-09/19	518-550	Contact Rate14	11.0781	Ashoora (Aug 18 to 19, 2021)	[89]
2021/09/19-12/18	550-640	Contact Rate15	10.0448	Eid Milad-un-Nabi (Oct 19, 2021)<br>Quaid-e-Azam Day (Dec 25, 2021)	[90,91]
2021/12/18-2022/01/25	640-678	Contact Rate16	14.1307	Omicron variant outbreak (First confirmed case: Dec 13, 2021)	[92]
2022/01/25-02/28	678-712	Contact Rate17	11.3083	Karachi Central Smart Lockdown (Jan 22 to Feb 5, 2022)<br>Restrictions on gatherings (Jan 20 to Feb 17, 2022)	[93,94]

Table S4. Calibrated Infectivity of symptomatic and asymptomatic cases in **Pakistan**

Actual date	Model time	Infectivity of symptomatic cases	Calibration Value	Infectivity of asymptomatic cases	Calibration Value
2020/03/18-03/23	0-5	Infectivity of symptomatic cases1	0.0330641	Infectivity of asymptomatic cases1	0.00698104
2020/03/23-05/09	5-52	Infectivity of symptomatic cases2	0.0313523	Infectivity of asymptomatic cases2	0.00801761
2020/05/09-06/16	52-90	Infectivity of symptomatic cases3	0.0219013	Infectivity of asymptomatic cases3	0.00599135
2020/06/16-06/30	90-104	Infectivity of symptomatic cases4	0.004	Infectivity of asymptomatic cases4	0.001
2020/06/30-08/29	104-164	Infectivity of symptomatic cases5	0.00481322	Infectivity of asymptomatic cases5	0.00180754
2020/08/29-11/13	164-240	Infectivity of symptomatic cases6	0.0131214	Infectivity of asymptomatic cases6	0.00481451
2020/11/13-12/21	240-278	Infectivity of symptomatic cases7	0.0242961	Infectivity of asymptomatic cases7	0.00671673
202012/21-2021/03/01	278-348	Infectivity of symptomatic cases8	0.0111058	Infectivity of asymptomatic cases8	0.0025632
2021/03/01-04/11	348-389	Infectivity of symptomatic cases9	0.0205673	Infectivity of asymptomatic cases9	0.00400045
2021/04/11-05/08	389-416	Infectivity of symptomatic cases10	0.00817915	Infectivity of asymptomatic cases10	0.00221594
2021/05/08-05/16	416-424	Infectivity of symptomatic cases11	0.00648643	Infectivity of asymptomatic cases11	0.00248971
2021/05/16-07/01	424-470	Infectivity of symptomatic cases12	0.00867988	Infectivity of asymptomatic cases12	0.00100252
2021/07/01-08/18	470-518	Infectivity of symptomatic cases13	0.0180911	Infectivity of asymptomatic cases13	0.00363475
2021/08/18-09/19	518-550	Infectivity of symptomatic cases14	0.0115929	Infectivity of asymptomatic cases14	0.00310669
2021/09/19-12/18	550-640	Infectivity of symptomatic cases15	0.0123267	Infectivity of asymptomatic cases15	0.00201239
2021/12/18-2022/01/25	640-678	Infectivity of symptomatic cases16	0.059228	Infectivity of asymptomatic cases16	0.0171305
2022/01/25-02/28	678-712	Infectivity of symptomatic cases17	0.00993202	Infectivity of asymptomatic cases17	0.00597904

Table S5. Calibrated death ratio in **Pakistan** (death over severe cases)

Actual date	Model time	Death ratio in hospitalization condition	Value	Death ratio in untreated condition	Value
2020/03/18-06/16	0-90	Death ratio1	0.12237	death ratio without treatment1	0.269997
2020/06/16-06/30	90-104	Death ratio2	0.069561	death ratio without treatment2	0.142723
2020/06/30-08/29	104-164	Death ratio3	0.0106682	death ratio without treatment3	0.0364157
2020/08/29-11/13	164-240	Death ratio4	0.0138719	death ratio without treatment4	0.032583
2020/11/13-12/21	240-278	Death ratio5	0.0999964	death ratio without treatment5	0.298732
202012/21-2021/03/01	278-348	Death ratio6	0.0376878	death ratio without treatment6	0.120713
2021/03/01-05/08	348-416	Death ratio7	0.0499132	death ratio without treatment7	0.116914
2020/05/08-07/01	416-470	Death ratio8	0.0219973	death ratio without treatment8	0.0562207
2021/07/01-09/19	470-550	Death ratio9	0.0439793	death ratio without treatment9	0.0904876
2021/09/19-12/18	550-640	Death ratio10	0.0146682	death ratio without treatment10	0.038327
2021/12/18-2022/02/28	640-712	Death ratio11	0.00998797	death ratio without treatment11	0.0219324

Table S6. Calibrated average contact rate at different period in **Bangladesh**

Actual date	Model time	Contact Rate	Calibrated value	Measures and social activity	Sources
2020/03/18-03/26	0-8	Contact Rate1	26.2249		
2020/03/26-05/30	8-73	Contact Rate2	11.9561	1st countrywide lockdown	[95]
2020/05/30-07/31	73-135	Contact Rate3	15.8669		
2020/07/31-09/04	135-170	Contact Rate4	13.1295	Eid al-Adha (July 31 to Aug 2, 2020)<br>National Mourning Day (Aug 15, 2020)	[96,97]
2020/09/04-10/14	170-210	Contact Rate5	12.8526		
2020/10/14-12/13	210-270	Contact Rate6	16.4593	Laxmi Pooja (Nov 14, 2020)	[98]
202012/13-2021/02/21	270-340	Contact Rate7	13.8023	Victory day of Bangladesh (Dec 16, 2020)	[99]
2021/02/21-04/05	340-383	Contact Rate8	16.0078	International Mother Language Day (Feb 21, 2021)<br>Sheikh Mujibur Rahman's Birth Anniversary (Mar 17, 2021)<br>Independence Day of Bangladesh (Mar 26, 2021)	[100-102]
2021/04/05-05/24	383-432	Contact Rate9	11.0002	2nd countrywide lockdown<br>Bengali New Year (Pôhela Boishakh) (Apr 14, 2021)<br>Eid al-Fitr (May 13 to 15, 2021)	[95,103,104]
2021/05/24-07/01	432-470	Contact Rate10	13.6028	Vesak (May 26, 2021)	[105]
2021/07/01-07/14	470-483	Contact Rate11	11.0117	3rd countrywide lockdown	[95]
2021/07/14-07/23	483-492	Contact Rate12	11.2066	Eid al-Adha (July 21 to 23, 2021)	[106]
2021/07/23-08/11	492-511	Contact Rate13	11.0014	4th countrywide lockdown	[95]
2021/08/11-12/13	511-635	Contact Rate14	11.0106	National Mourning Day (Aug 15, 2021)<br>Shuba Janmashtami (Aug 19, 2021)	[107,108]
2021/12/13-2022/01/22	635-675	Contact Rate15	17.7993	Omicron variant outbreak (First confirmed case: Dec 11, 2021)<br>International Mother Language Day (Feb 21, 2022)	[109,110]
2022/01/22-02/28	675-712	Contact Rate16	13.0032	Bangladesh notifies restrictions on public gathering, transport, restaurants (January 10 to Feb 21, 2022)	[111,112]

Table S7. Calibrated Infectivity of symptomatic and asymptomatic cases in **Bangladesh**

Actual date	Model time	Infectivity of symptomatic cases	Calibration Value	Infectivity of asymptomatic cases	Calibration Value
2020/03/18-03/26	0-8	Infectivity of symptomatic cases1	0.0340519	Infectivity of asymptomatic cases1	0.0099874
2020/03/26-05/30	8-73	Infectivity of symptomatic cases2	0.039058	Infectivity of asymptomatic cases2	0.0122406
2020/05/30-07/31	73-135	Infectivity of symptomatic cases3	0.0110313	Infectivity of asymptomatic cases3	0.00349587
2020/07/31-09/04	135-170	Infectivity of symptomatic cases4	0.00947761	Infectivity of asymptomatic cases4	0.0020297
2020/09/04-10/14	170-210	Infectivity of symptomatic cases5	0.0110139	Infectivity of asymptomatic cases5	0.00446161
2020/10/14-12/13	210-270	Infectivity of symptomatic cases6	0.0109675	Infectivity of asymptomatic cases6	0.00349955
202012/13-2021/02/21	270-340	Infectivity of symptomatic cases7	0.00652461	Infectivity of asymptomatic cases7	0.00130389
2021/02/21-04/05	340-383	Infectivity of symptomatic cases8	0.0261713	Infectivity of asymptomatic cases8	0.00606031
2021/04/05-05/24	383-432	Infectivity of symptomatic cases9	0.00740709	Infectivity of asymptomatic cases9	0.001
2021/05/24-07/01	432-470	Infectivity of symptomatic cases10	0.0209326	Infectivity of asymptomatic cases10	0.00715109
2021/07/01-07/14	470-483	Infectivity of symptomatic cases11	0.019577	Infectivity of asymptomatic cases11	0.00598135
2021/07/14-07/23	483-492	Infectivity of symptomatic cases12	0.004	Infectivity of asymptomatic cases12	0.001
2021/07/23-08/11	492-511	Infectivity of symptomatic cases13	0.0107985	Infectivity of asymptomatic cases13	0.00250728
2021/08/11-12/13	511-635	Infectivity of symptomatic cases14	0.004	Infectivity of asymptomatic cases14	0.001
2021/12/13-2022/01/22	635-675	Infectivity of symptomatic cases15	0.0636171	Infectivity of asymptomatic cases15	0.0177451
2022/01/22-02/28	675-712	Infectivity of symptomatic cases16	0.00951014	Infectivity of asymptomatic cases16	0.00467128

Table S8. Calibrated death ratio in **Bangladesh** (death over severe cases)

Actual date	Model time	Death ratio in hospitalization condition	Value	Death ratio in untreated condition	Value
2020/03/18-05/30	0-73	Death ratio1	0.115231	death ratio without treatment1	0.349947
2020/05/30-07/31	73-135	Death ratio2	0.0219848	death ratio without treatment2	0.0469918
2020/07/31-10/14	135-210	Death ratio3	0.0120023	death ratio without treatment3	0.0356461
2020/10/14-2021/02/21	210-340	Death ratio4	0.0186436	death ratio without treatment4	0.0375896
2021/02/21-04/05	340-383	Death ratio5	0.0359902	death ratio without treatment5	0.0605898
2021/04/05-07/01	383-470	Death ratio6	0.0180162	death ratio without treatment6	0.0513109
2021/07/01-07/23	470-492	Death ratio7	0.0211944	death ratio without treatment7	0.0906717
2020/07/23-08/11	492-511	Death ratio8	0.0341644	death ratio without treatment8	0.0522761
2021/08/11-09/04	511-535	Death ratio9	0.00716992	death ratio without treatment9	0.0167481
2021/09/04-12/13	535-635	Death ratio10	0.0050848	death ratio without treatment10	0.0072611
2021/12/13-2022/02/28	635-712	Death ratio11	0.00357136	death ratio without treatment11	0.0051217

S3 Scenario Design

Table S9-1. Settings for Pakistan's counterfactual analysis scenarios **presented** in main text (cont'd)

**Scenario setting** (Mar 18, 2020--Feb 28, 2022)		**Non-pharmaceutical containment measures**										Medical resources	**Vaccine administration Initial protocol**					
		1st lockdown		2nd lockdown		Added lockdown in the 2nd wave		Adding lockdown in the 4th wave		Adding lockdown in the 5th wave		Hospital capacity	1st dose Original avg. pace: 323240/day			2nd dose Original avg. pace: 264905/day		
Scenarios	Description	Setting change	Period Day5 -52	Setting change	Period Day416 -424	Setting change	Period	Setting change	Period	Setting change	Period		Setting change	Pace change	Period Day322 -714	Setting change	Pace change	Period Day340 -712
P1	Shifting imposing date of the 1st lockdown 5 days backward	✓	0-52	✘	N/A	✘	N/A	✘	N/A	✘	N/A	N/A	✘	✘	N/A	✘	✘	N/A
P2	Shifting lifting date of the 1st lockdown 1 week forward	✓	5-59	✘	N/A	✘	N/A	✘	N/A	✘	N/A	N/A	✘	✘	N/A	✘	✘	N/A
P3	Shifting lifting date of the 1st lockdown 2 weeks forward	✓	5-66	✘	N/A	✘	N/A	✘	N/A	✘	N/A	N/A	✘	✘	N/A	✘	✘	N/A
P4	Shifting lifting date of the 1st lockdown 3 weeks forward	✓	5-73	✘	N/A	✘	N/A	✘	N/A	✘	N/A	N/A	✘	✘	N/A	✘	✘	N/A
P5	Shifting lifting date of the 1st lockdown 4 weeks forward	✓	5-80	✘	N/A	✘	N/A	✘	N/A	✘	N/A	N/A	✘	✘	N/A	✘	✘	N/A
P6	Shifting imposing date of the 1st lockdown 5 days backward + shifting lifting date of the 1st lockdown 1 week forward	✓	0-59	✘	N/A	✘	N/A	✘	N/A	✘	N/A	N/A	✘	✘	N/A	✘	✘	N/A
P7	Shifting imposing date of the 2nd lockdown 2 weeks backward + shifting lifting date of the 2nd lockdown 2 weeks forward	✘	N/A	✓	402-438	✘	N/A	✘	N/A	✘	N/A	N/A	✘	✘	N/A	✘	✘	N/A
P8	Shifting imposing date of the 2nd lockdown 6 weeks backward + shifting lifting date of the 2nd lockdown 2 weeks forward	✘	N/A	✓	374-438	✘	N/A	✘	N/A	✘	N/A	N/A	✘	✘	N/A	✘	✘	N/A
P9	Adding 3 weeks lockdown at the beginning of upward slope of the 2nd wave	✘	N/A	✘	N/A	✓	233-254	✘	N/A	✘	N/A	N/A	✘	✘	N/A	✘	✘	N/A
P10	Adding 6 weeks lockdown at the beginning of upward slope of the 2nd wave	✘	N/A	✘	N/A	✓	233-275	✘	N/A	✘	N/A	N/A	✘	✘	N/A	✘	✘	N/A
P11	Adding 2 weeks lockdown on the upward slope of the 4th wave	✘	N/A	✘	N/A	✘	N/A	✓	482-496	✘	N/A	N/A	✘	✘	N/A	✘	✘	N/A
P12	Adding 4 weeks lockdown on the upward slope of the 4th wave	✘	N/A	✘	N/A	✘	N/A	✓	482-510	✘	N/A	N/A	✘	✘	N/A	✘	✘	N/A
P13	Adding 1 week lockdown at the beginning of upward slope of the 5th wave	✘	N/A	✘	N/A	✘	N/A	✘	N/A	✓	666-673	N/A	✘	✘	N/A	✘	✘	N/A
P14	Adding 2 weeks lockdown at the beginning of upward slope of the 5th wave	✘	N/A	✘	N/A	✘	N/A	✘	N/A	✓	666-680	N/A	✘	✘	N/A	✘	✘	N/A
P15	Shifting imposing date of the 1st lockdown 5 days backward + shifting imposing date of the 2nd lockdown 6 weeks backward	✓	0-52	✓	374-424	✘	N/A	✘	N/A	✘	N/A	N/A	✘	✘	N/A	✘	✘	N/A

Table S9-2. Settings for Pakistan's counterfactual analysis scenarios **presented** in main text (cont'd)

**Scenario setting** (Mar 18, 2020--Feb 28, 2022)		**Non-pharmaceutical containment measures**										**Medical resources**	**Vaccine administration Initial protocol**					
		1st lockdown		2nd lockdown		Added lockdown in the 2nd wave		Adding lockdown in the 4th wave		Adding lockdown in the 5th wave		Hospital capacity	1st dose Original avg. pace: 323240/day			2nd dose Original avg. pace: 264905/day		
Scenarios	Description	Setting change	Period Day5-52	Setting change	Period Day416-424	Setting change	Period	Setting change	Period	Setting change	Period		Setting change	Pace change	Period Day322-714	Setting change	Pace change	Period Day340-712
P16	Shifting imposing date of the 1st lockdown 5 days backward + shifting imposing date of the 2nd lockdown 6 weeks backward + adding 6 weeks lockdown at the beginning of upward slope of the 2nd wave + adding 4 weeks lockdown on the upward slope of the 4th wave	✓	0-52	✓	374-424	✓	233-275	✓	482-510	✘	N/A	N/A	✘	✘	N/A	✘	✘	N/A
P17	Shifting imposing date of the 1st lockdown 5 days backward + shifting imposing date of the 2nd lockdown 6 weeks backward + adding 6 weeks lockdown at the beginning of upward slope of the 2nd wave + adding 4 weeks lockdown on the upward slope of the 4th wave + adding 1 week lockdown at the beginning of the upward slope of the 5th wave	✓	0-52	✓	374-424	✓	233-275	✓	482-510	✓	666-673	N/A	✘	✘	N/A	✘	✘	N/A
P18	Shifting imposing date of the 1st lockdown 5 days backward + shifting lifting date of the 1st lockdown 4 weeks forward + shifting imposing date of the 2nd lockdown 4 weeks backward	✓	0-80	✓	388-424	✘	N/A	✘	N/A	✘	N/A	N/A	✘	✘	N/A	✘	✘	N/A
P19	Shifting imposing date of the 1st lockdown 5 days backward + shifting lifting date of the 1st lockdown 4 weeks forward + shifting imposing date of 2nd lockdown 6 weeks backward	✓	0-80	✓	374-424	✘	N/A	✘	N/A	✘	N/A	N/A	✘	✘	N/A	✘	✘	N/A
P20	Shifting imposing date of the 1st lockdown 5 days backward + shifting lifting date of the 1st lockdown 4 weeks forward + shifting imposing date of the 2nd lockdown 6 weeks backward + adding 6 weeks lockdown at the beginning of upward slope of 2nd wave	✓	0-80	✓	374-424	✓	233-275	✘	N/A	✘	N/A	N/A	✘	✘	N/A	✘	✘	N/A
P21	Shifting imposing date of the 1st lockdown 5 days backward + shifting lifting date of the 1st lockdown 4 weeks forward + shifting imposing date of the 2nd lockdown 6 weeks backward + adding 6 weeks lockdown at the beginning of upward slope of the 2nd wave + adding 4 weeks lockdown on the upward slope of the 4th wave	✓	0-80	✓	374-424	✓	233-275	✓	482-510	✘	N/A	N/A	✘	✘	N/A	✘	✘	N/A
P22	Increasing hospital capacity by 20%	✘	N/A	✘	N/A	✘	N/A	✘	N/A	✘	N/A	1.2 times	✘	✘	N/A	✘	✘	N/A

Table S9-3. Settings for Pakistan's counterfactual analysis scenarios **presented** in main text (cont'd)

**Scenario setting** (Mar 18, 2020--Feb 28, 2022)		**Non-pharmaceutical containment measures**										Medical resources	**Vaccine administration Initial protocol**					
		1st lockdown		2nd lockdown		Added lockdown in the 2nd wave		Adding lockdown in the 4th wave		Adding lockdown in the 5th wave			1st dose Original avg. pace: 323240/day			2nd dose Original avg. pace: 264905/day		
Scenarios	Description	Setting change	Period Day5-52	Setting change	Period Day416-424	Setting change	Period	Setting change	Period	Setting change	Period	Hospital capacity	Setting change	Pace change	Period Day322-714	Setting change	Pace change	Period Day340-712
P23	Increasing hospital capacity by 50%	✓	N/A	✘	N/A	✘	N/A	✘	N/A	✘	N/A	1.5times	✘	✘	N/A	✘	✘	N/A
P24	Shifting lifting date of the 1st lockdown 1 weeks forward + increasing hospital capacity by 20%	✘	5-59	✘	N/A	✘	N/A	✘	N/A	✘	N/A	1.2 times	✘	✘	N/A	✘	✘	N/A
P25	Shifting imposing date of the 1st lockdown 5 days backward + shifting lifting date of the 1st lockdown 4 weeks forward + shifting imposing date of the 2nd lockdown 4 weeks backward + increasing hospital capacity by 20%	✓	0-80	✓	388-424	✘	N/A	✘	N/A	✘	N/A	1.2 times	✘	✘	N/A	✘	✘	N/A
P26	Shifting imposing date of the 1st lockdown 5 days backward + shifting lifting date of the 1st lockdown 4 weeks forward + shifting imposing date of the 2nd lockdown 6 weeks backward + increasing hospital capacity by 20%	✓	0-80	✓	374-424	✘	N/A	✘	N/A	✘	N/A	1.2 times	✘	✘	N/A	✘	✘	N/A
P27	Shifting imposing date of the 1st lockdown 5 days backward + shifting lifting date of the 1st lockdown 4 weeks forward + shifting imposing date of the 2nd lockdown 6 weeks backward + adding 6 weeks lockdown at the beginning of upward slope of 2nd wave + increasing hospital capacity by 20%	✓	0-80	✓	374-424	✓	233-275	✘	N/A	✘	N/A	1.2 times	✘	✘	N/A	✘	✘	N/A
P28	One month early-launching of the 1st and the 2nd dose vaccine administration	✘	N/A	✘	N/A	✘	N/A	✘	N/A	✘	N/A	N/A	✓	✘	291-712	✓	✘	309-712
P29	Two months early-launching the 1st and the 2nd dose vaccine administration	✘	N/A	✘	N/A	✘	N/A	✘	N/A	✘	N/A	N/A	✓	✘	260-712	✓	✘	278-712
P30	Increasing vaccination pace 100%	✘	N/A	✘	N/A	✘	N/A	✘	N/A	✘	N/A	N/A	✓	Twice	N/A	✓	Twice	N/A
P31	Two months early-launching the 1st and the 2nd dose vaccine administration + Increasing vaccination pace 100%	✘	N/A	✘	N/A	✘	N/A	✘	N/A	✘	N/A	N/A	✓	Twice	260-712	✓	Twice	278-712
P32	Shifting imposing date of 1st lockdown 5 days backward + shifting imposing date of the 2nd lockdown 1 weeks backward + Two month early-launching the 1st and the 2nd dose vaccine administration + Increasing vaccination pace 50%	✓	0-52	✓	409-424	✘	N/A	✘	N/A	✘	N/A	N/A	✓	1.5 times	260-712	✓	1.5 times	278-712

Table S9-4. Settings for Pakistan's counterfactual analysis scenarios **presented** in main text

**Scenario setting** (Mar 18, 2020--Feb 28, 2022)		**Non-pharmaceutical containment measures**										Medical resources	**Vaccine administration Initial protocol**					
		1st lockdown		2nd lockdown		Added lockdown in the 2nd wave		Adding lockdown in the 4th wave		Adding lockdown in the 5th wave		Hospital capacity	1st dose Original avg. pace: 323240/day			2nd dose Original avg. pace: 264905/day		
Scenarios	Description	Setting change	Period Day5 -52	Setting change	Period Day416 -424	Setting change	Period	Setting change	Period	Setting change	Period		Setting change	Pace change	Period Day322 -714	Setting change	Pace change	Period Day340 -712
P33	Shifting imposing date of the 1st lockdown 5 days backward + shifting imposing date of the 2nd lockdown 1 week backward + Two month early-launching the 1st and the 2nd dose vaccine administration + Increasing vaccination pace 100%	✓	0-52	✓	409-424	✘	N/A	✘	N/A	✘	N/A	N/A	✓	Twice	260-712	✓	Twice	278-712
P34	Shifting imposing date of the 1st lockdown 5 days backward + Shifting lifting date of the 1st lockdown 4 weeks forward + shifting imposing date of the 2nd lockdown 6 weeks backward + adding 6 weeks lockdown at the beginning of upward slope of the 2nd wave+ Two month early-launching the 1st and the 2nd dose vaccine administration + Increasing vaccination pace 100%	✓	0-80	✓	374-424	✘	N/A	✘	N/A	✘	N/A	N/A	✓	Twice	260-712	✓	Twice	278-712

Table S10-1. Settings for Pakistan's counterfactual analysis scenarios **not presented** in main text(cont'd)

**Scenario setting** (Mar 18, 2020--Feb 28, 2022)		**Non-pharmaceutical containment measures**										Medical resources	**Vaccine administration Initial protocol**					
		1st lockdown		2nd lockdown		Added lockdown in the 2nd wave		Adding lockdown in the 4th wave		Adding lockdown in the 5th wave		Hospital capacity	1st dose Original avg. pace: 323240/day			2nd dose Original avg. pace: 264905/day		
Scenarios	Description	Setting change	Period Day5 -52	Setting change	Period Day416 -424	Setting change	Period	Setting change	Period	Setting change	Period		Setting change	Pace chang e	Period Day322 -714	Settin g chang e	Pace chang e	Period Day340 -712
PA1	Shifting imposing date of the 1st lockdown 5 days backward + shifting lifting date of the 1st lockdown 2 weeks forward	✓	0-66	✘	N/A	✘	N/A	✘	N/A	✘	N/A	N/A	✘	✘	N/A	✘	✘	N/A
PA2	Shifting imposing date of the 1st lockdown 5 days backward + shifting lifting date of the 1st lockdown 3 weeks forward	✓	0-73	✘	N/A	✘	N/A	✘	N/A	✘	N/A	N/A	✘	✘	N/A	✘	✘	N/A
PA3	Shifting imposing date of the 1st lockdown 5 days backward + shifting lifting date of the 1st lockdown 4 weeks forward	✓	0-80	✘	N/A	✘	N/A	✘	N/A	✘	N/A	N/A	✘	✘	N/A	✘	✘	N/A
PA4	Shifting imposing date of the 2nd lockdown 1 week backward	✘	N/A	✓	409-424	✘	N/A	✘	N/A	✘	N/A	N/A	✘	✘	N/A	✘	✘	N/A
PA5	Shifting imposing date of the 2nd lockdown 1 week backward + shifting lifting date of the 2nd lockdown 1 week backward	✘	N/A	✓	409-417	✘	N/A	✘	N/A	✘	N/A	N/A	✘	✘	N/A	✘	✘	N/A
PA6	Shifting imposing date of the 2nd lockdown 1 week backward + shifting lifting date of the 2nd lockdown 1 week forward	✘	N/A	✓	409-431	✘	N/A	✘	N/A	✘	N/A	N/A	✘	✘	N/A	✘	✘	N/A
PA7	Shifting imposing date of the 2nd lockdown 1 week backward + shifting lifting date of the 2nd lockdown 2 weeks forward	✘	N/A	✓	409-438	✘	N/A	✘	N/A	✘	N/A	N/A	✘	✘	N/A	✘	✘	N/A
PA8	Shifting imposing date of the 2nd lockdown 2 weeks backward	✘	N/A	✓	402-424	✘	N/A	✘	N/A	✘	N/A	N/A	✘	✘	N/A	✘	✘	N/A
PA9	Shifting imposing date of the 2nd lockdown 2 weeks backward + shifting lifting date of the 2nd lockdown 2 weeks backward	✘	N/A	✓	402-410	✘	N/A	✘	N/A	✘	N/A	N/A	✘	✘	N/A	✘	✘	N/A
PA10	Shifting imposing date of the 2nd lockdown 3 week backward	✘	N/A	✓	395-424	✘	N/A	✘	N/A	✘	N/A	N/A	✘	✘	N/A	✘	✘	N/A
PA11	Shifting imposing date of the 2nd lockdown 3 weeks backward + shifting lifting date of the 2nd lockdown 3 weeks backward	✘	N/A	✓	395-403	✘	N/A	✘	N/A	✘	N/A	N/A	✘	✘	N/A	✘	✘	N/A
PA12	Shifting imposing date of the 2nd lockdown 3 weeks backward + shifting lifting date of the 2nd lockdown 2 weeks forward	✘	N/A	✓	395-438	✘	N/A	✘	N/A	✘	N/A	N/A	✘	✘	N/A	✘	✘	N/A
PA13	Shifting imposing date of the 2nd lockdown 4 weeks backward	✘	N/A	✓	388-424	✘	N/A	✘	N/A	✘	N/A	N/A	✘	✘	N/A	✘	✘	N/A
PA14	Shifting imposing date of the 2nd lockdown 6 weeks backward	✘	N/A	✓	374-424	✘	N/A	✘	N/A	✘	N/A	N/A	✘	✘	N/A	✘	✘	N/A
PA15	Adding 1 week lockdown at the beginning of upward slope of the 2nd wave	✘	N/A	✘	N/A	✓	233-240	✘	N/A	✘	N/A	N/A	✘	✘	N/A	✘	✘	N/A
PA16	Adding 2 weeks lockdown at the beginning of upward slope of the 2nd wave	✘	N/A	✘	N/A	✓	233-247	✘	N/A	✘	N/A	N/A	✘	✘	N/A	✘	✘	N/A

Table S10-2. Settings for Pakistan's counterfactual analysis scenarios **not presented** in main text(cont'd)

Scenario setting (Mar 18, 2020--Feb 28, 2022)		**Non-pharmaceutical containment measures**										Medical resources	**Vaccine administration Initial protocol**					
		1st lockdown		2nd lockdown		Added lockdown in the 2nd wave		Adding lockdown in the 4th wave		Adding lockdown in the 5th wave		Hospital capacity	1st dose Original avg. pace: 323240/day			2nd dose Original avg. pace: 264905/day		
Scenarios	Description	Setting change	Period Day5-52	Setting change	Period Day416-424	Setting change	Period	Setting change	Period	Setting change	Period		Setting change	Pace change	Period Day322-714	Setting change	Pace change	Period Day340-712
PA17	Adding 4 weeks lockdown at the beginning of upward slope of the 2nd wave	✘	N/A	✘	N/A	✓	233-261	✘	N/A	✘	N/A	N/A	✘	✘	N/A	✘	✘	N/A
PA18	Adding 1 week lockdown on the upward slope of the 4th wave	✘	N/A	✘	N/A	✘	N/A	P	482-489	✘	N/A	N/A	✘	✘	N/A	✘	✘	N/A
PA19	Adding 3 weeks lockdown on the upward slope of the 4th wave	✘	N/A	✘	N/A	✘	N/A	P	482-503	✘	N/A	N/A	✘	✘	N/A	✘	✘	N/A
PA20	Shifting imposing date of the 1st lockdown 5 days backward + shifting imposing date of the 2nd lockdown 1 week backward	✓	0-52	✓	409-424	✘	N/A	✘	N/A	✘	N/A	N/A	✘	✘	N/A	✘	✘	N/A
PA21	Shifting imposing date of the 1st lockdown 5 days backward + shifting imposing date of the 2nd lockdown 4 weeks backward	✓	0-52	✓	388-424	✘	N/A	✘	N/A	✘	N/A	N/A	✘	✘	N/A	✘	✘	N/A
PA22	Shifting imposing date of the 1st lockdown 5 days backward + shifting imposing date of the 2nd lockdown 1 week backward + adding 1 week lockdown at the beginning of upward slope of the 2nd wave	✓	0-52	✓	409-424	✓	233-240	✘	N/A	✘	N/A	N/A	✘	✘	N/A	✘	✘	N/A
PA23	Shifting imposing date of the 1st lockdown 5 days backward + shifting imposing date of the 2nd lockdown 1 week backward + adding 1 week lockdown at the beginning of upward slope of the 2nd wave + adding 1 week lockdown on the upward slope of the 4th wave	✓	0-52	✓	409-424	✓	233-240	P	482-489	✘	N/A	N/A	✘	✘	N/A	✘	✘	N/A
PA24	Shifting imposing date of the 1st lockdown 5 days backward + shifting imposing date of the 2nd lockdown 2 weeks backward + adding 2 weeks lockdown at the beginning of upward slope of the 2nd wave + adding 2 weeks lockdown on the upward slope of the 4th wave	✓	0-52	✓	402-424	✓	233-247	✓	482-496	✘	N/A	N/A	✘	✘	N/A	✘	✘	N/A
PA25	Shifting imposing date of the 1st lockdown 5 days backward + shifting lifting date of the 1st lockdown 4 weeks forward + shifting imposing date of the 2nd lockdown 4 weeks backward + adding 4 weeks lockdown at the beginning of upward slope of the 2nd wave	✓	0-80	✓	388-424	✓	233-261	✘	N/A	✘	N/A	N/A	✘	✘	N/A	✘	✘	N/A

Table S10-3. Settings for Pakistan's counterfactual analysis scenarios **not presented** in main text(cont'd)

**Scenario setting** (Mar 18, 2020--Feb 28, 2022)		**Non-pharmaceutical containment measures**										Medical resources	**Vaccine administration Initial protocol**					
		1st lockdown		2nd lockdown		Added lockdown in the 2nd wave		Adding lockdown in the 4th wave		Adding lockdown in the 5th wave		Hospital capacity	1st dose Original avg. pace: 323240/day			2nd dose Original avg. pace: 264905/day		
Scenarios	Description	Setting change	Period Day5-52	Setting change	Period Day416-424	Setting change	Period	Setting change	Period	Setting change	Period		Setting change	Pace change	Period Day322-714	Setting change	Pace change	Period Day340-712
PA26	Shifting imposing date of the 1st lockdown 5 days backward + shifting lifting date of the 1st lockdown 4 weeks forward + shifting imposing date of the 2nd lockdown 6 weeks backward + adding 4 weeks lockdown at the beginning of upward slope of 2nd wave + adding 2 weeks lockdown on the upward slope of 4th wave	✓	0-80	✓	388-424	✓	233-261	✓	482-496	✘	N/A	N/A	✘	✘	N/A	✘	✘	N/A
PA27	Shifting imposing date of the 1st lockdown 5 days backward + shifting lifting date of the 1st lockdown 4 weeks forward + shifting imposing date of the 2nd lockdown 6 weeks backward + adding 4 weeks lockdown on the upward slope of the 4th wave	✓	0-80	✓	374-424	No	N/A	✓	482-510	✘	N/A	N/A	✘	✘	N/A	✘	✘	N/A
PA28	Increasing vaccination pace 50%	✘	N/A	✘	N/A	✘	N/A	✘	N/A	✘	N/A	N/A	✓	1.5 times	N/A	✓	1.5 times	N/A
PA29	Increasing vaccination pace 80%	✘	N/A	✘	N/A	✘	N/A	✘	N/A	✘	N/A	N/A	✓	1.8 times	N/A	✓	1.8 times	N/A
PA30	One month early-launching the 1st and the 2nd dose vaccine administration + Increasing vaccination pace 50%	✘	N/A	✘	N/A	✘	N/A	✘	N/A	✘	N/A	N/A	✓	1.5 times	291-712	✓	1.5 times	309-712
PA31	One month early-launching the 1st and the 2nd dose vaccine administration + Increasing vaccination pace 80%	✘	N/A	✘	N/A	✘	N/A	✘	N/A	✘	N/A	N/A	✓	1.8 times	291-712	✓	1.8 times	309-712
PA32	One month early-launching the 1st and the 2nd dose vaccine administration + Increasing vaccination pace 100%	✘	N/A	✘	N/A	✘	N/A	✘	N/A	✘	N/A	N/A	✓	Twice	291-712	✓	Twice	309-712
PA33	Two months early-launching the 1st and the 2nd dose vaccine administration + Increasing vaccination pace 50%	✘	N/A	✘	N/A	✘	N/A	✘	N/A	✘	N/A	N/A	✓	1.5 times	260-712	✓	1.5 times	278-712
PA34	Two months early-launching the 1st and the 2nd dose vaccine administration + Increasing vaccination pace 80%	✘	N/A	✘	N/A	✘	N/A	✘	N/A	✘	N/A	N/A	✓	1.8 times	260-712	✓	1.8 times	278-712
PA35	Shifting imposing date of the 1st lockdown 5 days backward + Shifting imposing date of the 2nd lockdown 1 weeks backward + Increasing vaccination pace 50%	✓	0-52	✓	409-424	✘	N/A	✘	N/A	✘	N/A	N/A	✓	1.5 times	N/A	✓	1.5 times	N/A

Table S10-4. Settings for Pakistan's counterfactual analysis scenarios **not presented** in main text

**Scenario setting** (Mar 18, 2020--Feb 28, 2022)		**Non-pharmaceutical containment measures**										Medical resources	**Vaccine administration Initial protocol**					
		1st lockdown		2nd lockdown		Added lockdown in the 2nd wave		Adding lockdown in the 4th wave		Adding lockdown in the 5th wave			1st dose Original avg. pace: 323240/day			2nd dose Original avg. pace: 264905/day		
Scenarios	Description	Setting change	Period Day5-52	Setting change	Period Day416-424	Setting change	Period	Setting change	Period	Setting change	Period	Hospital capacity	Setting change	Pace change	Period Day322-714	Setting change	Pace change	Period Day340-712
PA36	Shifting imposing date of the 1st lockdown 5 days backward + Shifting imposing date of the 2nd lockdown 1 weeks backward + Increasing vaccination pace 100%	✓	0-52	✓	409-424	✘	N/A	✘	N/A	✘	N/A	N/A	✓	Twice	N/A	✓	Twice	N/A
PA37	Shifting imposing date of the 1st lockdown 5 days backward + shifting lifting date of the 1st lockdown 4 weeks forward + Shifting imposing date of the 2nd lockdown 4 weeks backward + Increasing vaccination pace 100%	✓	0-80	✓	388-424	✘	N/A	✘	N/A	✘	N/A	N/A	✓	Twice	N/A	✓	Twice	N/A
PA38	Shifting imposing date of the 1st lockdown 5 days backward + shifting lifting date of the 1st lockdown 4 weeks forward + Shifting imposing date of the 2nd lockdown 6 weeks backward + Increasing vaccination pace 100%	✓	0-80	✓	374-424	✘	N/A	✘	N/A	✘	N/A	N/A	✓	Twice	N/A	✓	Twice	N/A
PA39	Shifting imposing date of the 1st lockdown 5 days backward + Shifting lifting date of the 1st lockdown 4 weeks forward + shifting imposing date of the 2nd lockdown 6 weeks backward + adding 6 weeks lockdown at the beginning of upward slope of the 2nd wave + Increasing vaccination pace 100%	✓	0-80	✓	374-424	✓	233-275	✘	N/A	✘	N/A	N/A	✓	Twice	N/A	✓	Twice	N/A
PA40	Shifting imposing date of the 1st lockdown 5 days backward + Shifting lifting date of the 1st lockdown 4 weeks forward + shifting imposing date of the 2nd lockdown 6 weeks backward + adding 6 weeks lockdown at the beginning of upward slope of the 2nd wave + One month early-launching the 1st and the 2nd dose vaccine administration + Increasing vaccination pace 50%	✓	0-80	✓	374-424	✓	233-275	✘	N/A	✘	N/A	N/A	✓	1.5 times	291-712	✓	1.5 times	309-712
PA41	Shifting imposing date of the 1st lockdown 5 days backward + Shifting lifting date of the 1st lockdown 4 weeks forward + shifting imposing date of the 2nd lockdown 6 weeks backward + adding 6 weeks lockdown at the beginning of upward slope of the 2nd wave + Two month early-launching the 1st and the 2nd dose vaccine administration + Increasing vaccination pace 50%	✓	0-80	✓	374-424	✓	233-275	✘	N/A	✘	N/A	N/A	✓	1.5 times	260-712	✓	1.5 times	278-712
PA42	Shifting imposing date of the 1st lockdown 5 days backward + Shifting lifting date of the 1st lockdown 4 weeks forward + shifting imposing date of the 2nd lockdown 4 weeks backward + Two month early-launching the 1st and the 2nd dose vaccine administration + Increasing vaccination pace 100%	✓	0-80	✓	388-424	✘	N/A	✘	N/A	✘	N/A	N/A	✓	Twice	260-712	✓	Twice	278-712

Table S11-1. Settings for Bangladesh's counterfactual analysis scenarios **presented** in main text(cont'd)

**Scenario setting** (Mar 18, 2020--Feb 28, 2022)		**Non-pharmaceutical containment measures**												Medical resources	**Vaccine administration Initial protocol**					
		1st lockdown		2nd lockdown		3rd lockdown		4th lockdown		Added lockdown in the 2nd wave		Adding lockdown in the 5th wave			1st dose Original avg. pace: 310599/day			2nd dose Original avg. pace: 255186/day		
Scenarios	Description	Setting change	Period Day8-73	Setting change	Period Day383-432	Setting change	Period Day470-483	Setting change	Period Day492-511	Setting change	Period	Setting change	Period	Hospital capacity	Setting change	Pace change	Period Day315-712	Setting change	Pace change	Period Day386-712
B1	Shifting imposing date of the 1st lockdown 5 days backward	✓	3-73	✘	N/A	✘	N/A	✘	N/A	✘	N/A	✘	N/A	N/A	✘	✘	N/A	✘	✘	N/A
B2	Shifting lifting date of the 1st lockdown 1 week forward	✓	8-80	✘	N/A	✘	N/A	✘	N/A	✘	N/A	✘	N/A	N/A	✘	✘	N/A	✘	✘	N/A
B3	Shifting lifting date of the 1st lockdown 2 weeks forward	✓	8-87	✘	N/A	✘	N/A	✘	N/A	✘	N/A	✘	N/A	N/A	✘	✘	N/A	✘	✘	N/A
B4	Shifting lifting date of the 1st lockdown 3 weeks forward	✓	8-94	✘	N/A	✘	N/A	✘	N/A	✘	N/A	✘	N/A	N/A	✘	✘	N/A	✘	✘	N/A
B5	Shifting imposing date of the 1st lockdown 5 days backward + shifting lifting date of the 1st lockdown 3 weeks forward	✓	3-94	✘	N/A	✘	N/A	✘	N/A	✘	N/A	✘	N/A	N/A	✘	✘	N/A	✘	✘	N/A
B6	Shifting imposing date of the 2nd lockdown 1 week backward	✘	N/A	✓	376-432	✘	N/A	✘	N/A	✘	N/A	✘	N/A	N/A	✘	✘	N/A	✘	✘	N/A
B7	Shifting imposing date of the 2nd lockdown 2 weeks backward	✘	N/A	✓	369-432	✘	N/A	✘	N/A	✘	N/A	✘	N/A	N/A	✘	✘	N/A	✘	✘	N/A
B8	Shifting imposing date of the 2nd lockdown 2 weeks backward + shifting lifting date of the 2nd lockdown 2 weeks backward	✘	N/A	✓	369-418	✘	N/A	✘	N/A	✘	N/A	✘	N/A	N/A	✘	✘	N/A	✘	✘	N/A
B9	Shifting imposing date of the 2nd lockdown 2 weeks backward + shifting lifting date of the 2nd lockdown 2 weeks forward	✘	N/A	✓	369-446	✘	N/A	✘	N/A	✘	N/A	✘	N/A	N/A	✘	✘	N/A	✘	✘	N/A
B10	Shifting imposing date of the 3rd lockdown 1 week backward	✘	N/A	✘	N/A	✓	463-483	✘	N/A	✘	N/A	✘	N/A	N/A	✘	✘	N/A	✘	✘	N/A
B11	Shifting imposing date of the 3rd lockdown 2 weeks backward	✘	N/A	✘	N/A	✓	456-483	✘	N/A	✘	N/A	✘	N/A	N/A	✘	✘	N/A	✘	✘	N/A
B12	Adding 2 weeks lockdown at the beginning of upward slope of the 2nd wave	✘	N/A	✘	N/A	✘	N/A	✘	N/A	✓	242-256	✘	N/A	N/A	✘	✘	N/A	✘	✘	N/A
B13	Adding 4 weeks lockdown at the beginning of upward slope of the 2nd wave	✘	N/A	✘	N/A	✘	N/A	✘	N/A	✓	242-270	✘	N/A	N/A	✘	✘	N/A	✘	✘	N/A
B14	Adding 1 week lockdown at the beginning of upward slope of the 5th wave	✘	N/A	✘	N/A	✘	N/A	✘	N/A	✘	N/A	✓	666-673	N/A	✘	✘	N/A	✘	✘	N/A
B15	Adding 2 weeks lockdown at the beginning of upward slope of the 5th wave	✘	N/A	✘	N/A	✘	N/A	✘	N/A	✘	N/A	✓	666-680	N/A	✘	✘	N/A	✘	✘	N/A

Table S11-2. Settings for Bangladesh's counterfactual analysis scenarios **presented** in main text(cont'd)

**Scenario setting** (Mar 18, 2020--Feb 28, 2022)		**Non-pharmaceutical containment measures**												Medical resources	**Vaccine administration Initial protocol**					
		1st lockdown		2nd lockdown		3rd lockdown		4th lockdown		Added lockdown in the 2nd wave		Adding lockdown in the 5th wave			1st dose Original avg. pace: 310599/day			2nd dose Original avg. pace: 255186/day		
Scenarios	Description	Setting change	Period Day8-73	Setting change	Period Day383-432	Setting change	Period Day470-483	Setting change	Period Day492-511	Setting change	Period	Setting change	Period	Hospital capacity	Setting change	Pace change	Period Day315-712	Setting change	Pace change	Period Day386-712
B16	Shifting imposing date of the 1st lockdown 5 days backward + Shifting lifting date of the 1st lockdown 3 weeks forward + Cancelling the 2nd, 3rd, and 4th lockdowns	✓	3-94	✓	Cancelling	✓	Cancelling	✓	Cancelling	✘	N/A	✘	N/A	N/A	✘	✘	N/A	✘	✘	N/A
B17	Shifting imposing date of the 1st lockdown 5 days backward + Shifting lifting date of the 1st lockdown 3 weeks forward + Shifting imposing date of the 2nd lockdown 2 weeks backward + Cancelling the 3rd and 4th lockdowns	✓	3-94	✓	369-432	✓	Cancelling	✓	Cancelling	✘	N/A	✘	N/A	N/A	✘	✘	N/A	✘	✘	N/A
B18	Shifting imposing date of the 1st lockdown 5 days backward + Shifting imposing date of the 2nd lockdown 1 week backward + Shifting imposing date of the 3rd lockdown 1 week backward	✓	3-73	✓	376-432	✓	463-483	✘	N/A	✘	N/A	✘	N/A	N/A	✘	✘	N/A	✘	✘	N/A
B19	Shifting imposing date of the 1st lockdown 5 days backward + Shifting imposing date of the 2nd lockdown 2 weeks backward + Shifting imposing date of the 3rd lockdown 2 weeks backward	✓	3-73	✓	369-432	✓	456-483	✘	N/A	✘	N/A	✘	N/A	N/A	✘	✘	N/A	✘	✘	N/A
B20	Shifting imposing date of the 1st lockdown 5 days backward + Shifting lifting date of the 1st lockdown 3 weeks forward + Shifting imposing date of the 2nd lockdown 2 weeks backward + Shifting imposing date of the 3rd lockdown 2 weeks backward	✓	3-94	✓	369-432	✓	456-483	✘	N/A	✘	N/A	✘	N/A	N/A	✘	✘	N/A	✘	✘	N/A

Table S11-3. Settings for Bangladesh's counterfactual analysis scenarios **presented** in main text(cont'd)

Scenario setting (Mar 18, 2020--Feb 28, 2022)		Non-pharmaceutical containment measures												Medical resources	Vaccine administration Initial protocol					
		1st lockdown		2nd lockdown		3rd lockdown		4th lockdown		Added lockdown in the 2nd wave		Adding lockdown in the 5th wave			1st dose Original avg. pace: 310599/day			2nd dose Original avg. pace: 255186/day		
Scenarios	Description	Setting change	Period Day8-73	Setting change	Period Day383-432	Setting change	Period Day470-483	Setting change	Period Day492-511	Setting change	Period	Setting change	Period	Hospital capacity	Setting change	Pace change	Period Day315-712	Setting change	Pace change	Period Day386-712
B21	Shifting imposing date of the 1st lockdown 5 days backward + Shifting lifting date of the 1st lockdown 3 weeks forward + Shifting imposing date of the 2nd lockdown 2 weeks backward + Shifting lifting date of the 2nd lockdown 2 weeks forward + Shifting imposing date of the 3rd lockdown 2 weeks backward	✓	3-94	✓	369-446	✓	456-483	✘	N/A	✘	N/A	✘	N/A	N/A	✘	✘	N/A	✘	✘	N/A
B22	Shifting imposing date of the 1st lockdown 5 days backward + Shifting lifting date of the 1st lockdown 3 weeks forward + Shifting imposing date of the 2nd lockdown 2 weeks backward + Shifting imposing date of the 3rd lockdown 2 weeks backward + Adding 2 weeks lockdown at the beginning of upward slope of the 2nd wave	✓	3-94	✓	369-432	✓	456-483	✘	N/A	P	242-256	✘	N/A	N/A	✘	✘	N/A	✘	✘	N/A
B23	Increasing hospital capacity by 20%	✘	N/A	✘	N/A	✘	N/A	✘	N/A	✘	N/A	✘	N/A	1.2 times	✘	✘	N/A	✘	✘	N/A
B24	Increasing hospital capacity by 50%	✘	N/A	✘	N/A	✘	N/A	✘	N/A	✘	N/A	✘	N/A	1.5 times	✘	✘	N/A	✘	✘	N/A
B25	Shifting lifting date of the 1st lockdown 1 week forward + Increasing hospital capacity by 20%	✓	8-80	✘	N/A	✘	N/A	✘	N/A	✘	N/A	✘	N/A	1.2 times	✘	✘	N/A	✘	✘	N/A
B26	Shifting imposing date of the 1st lockdown 5 days backward + Shifting imposing date of the 2nd lockdown 1 week backward + Shifting imposing date of the 3rd lockdown 1 week backward + Increasing hospital capacity by 20%	✓	3-73	P	376-432	✓	463-483	✘	N/A	✘	N/A	✘	N/A	1.2 times	✘	✘	N/A	✘	✘	N/A

Table S11-4. Settings for Bangladesh's counterfactual analysis scenarios **presented** in main text

**Scenario setting** (Mar 18, 2020--Feb 28, 2022)				**Non-pharmaceutical containment measures**										Medical resources	**Vaccine administration Initial protocol**					
		1st lockdown		2nd lockdown		3rd lockdown		4th lockdown		Added lockdown in the 2nd wave		Adding lockdown in the 5th wave		Hospital capacity	1st dose Original avg. pace: 310599/day			2nd dose Original avg. pace: 255186/day		
Scenarios	Description	Setting change	Period Day8-73	Setting change	Period Day383-432	Setting change	Period Day470-483	Setting change	Period Day492-511	Setting change	Period	Setting change	Period		Setting change	Pace change	Period Day315-712	Setting change	Pace change	Period Day386-712
B27	Shifting imposing date of the 1st lockdown 5 days backward + Shifting lifting date of the 1st lockdown 3 weeks forward + Shifting imposing date of the 2nd lockdown 2 weeks backward + Shifting imposing date of the 3rd lockdown 2 weeks backward + Increasing hospital capacity by 20%	✓	3-94	✓	369-432	✓	456-483	✘	N/A	✘	N/A	✘	N/A	1.2 times	✘	✘	N/A	✘	✘	N/A
B28	Shifting imposing date of the 1st lockdown 5 days backward + Shifting lifting date of the 1st lockdown 3 weeks forward + Shifting imposing date of the 2nd lockdown 2 weeks backward + Shifting imposing date of the 3rd lockdown 2 weeks backward + Adding 2 weeks lockdown at the beginning of upward slope of the 2nd wave + Increasing hospital capacity by 20%	✓	3-94	✓	369-432	✓	456-483	✘	N/A	✓	242-256	✘	N/A	1.2 times	✘	✘	N/A	✘	✘	N/A
B29	One month early-launching the 1st and the 2nd dose vaccine administration	✘	N/A	✘	N/A	✘	N/A	✘	N/A	✘	N/A	✘	N/A	N/A	✓	✘	284-712	✓	✘	355-712
B30	Two months early-launching the 1st and the 2nd dose vaccine administration	✘	N/A	✘	N/A	✘	N/A	✘	N/A	✘	N/A	✘	N/A	N/A	✓	✘	254-712	✓	✘	327-712
B31	Increasing vaccination pace 100%	✘	N/A	✘	N/A	✘	N/A	✘	N/A	✘	N/A	✘	N/A	N/A	✓	Twice	N/A	✓	Twice	N/A
B32	Two months early-launching the 1st and the 2nd dose vaccine administration + Increasing vaccination pace 100%	✘	N/A	✘	N/A	✘	N/A	✘	N/A	✘	N/A	✘	N/A	N/A	✓	Twice	254-712	✓	Twice	327-712

Table S11-5. Settings for Bangladesh's counterfactual analysis scenarios **presented** in main text

**Scenario setting** (Mar 18, 2020--Feb 28, 2022)		**Non-pharmaceutical containment measures**												Medical resources	**Vaccine administration Initial protocol**					
		1st lockdown		2nd lockdown		3rd lockdown		4th lockdown		Added lockdown in the 2nd wave		Adding lockdown in the 5th wave			1st dose Original avg. pace: 310599/day			2nd dose Original avg. pace: 255186/day		
Scenarios	Description	Setting change	Period Day8-73	Setting change	Period Day383-432	Setting change	Period Day470-483	Setting change	Period Day492-511	Setting change	Period	Setting change	Period	Hospital capacity	Setting change	Pace change	Period Day315-712	Setting change	Pace change	Period Day386-712
B33	Shifting imposing date of the 1st lockdown 5 days backward + Shifting imposing date of the 2nd lockdown 1 week backward + Shifting imposing date of the 3rd lockdown 1 week backward + Two months early-launching the 1st and the 2nd dose vaccine administration + Increasing vaccination pace 50%	✓	3-73	✓	376-432	✓	463-483	✘	N/A	✓	N/A	✘	N/A	N/A	✓	1.5 times	254-712	✓	1.5 times	327-712
B34	Shifting imposing date of the 1st lockdown 5 days backward + Shifting lifting date of the 1st lockdown 3 weeks forward + Shifting imposing date of the 2nd lockdown 2 weeks backward + Shifting imposing date of the 3rd lockdown 2 weeks backward + Adding 2 weeks lockdown at the beginning of upward slope of the 2nd wave + Two months early-launching the 1st and the 2nd dose vaccine administration + Increasing vaccination pace 100%	✓	3-94	✓	369-432	✓	456-483	✘	N/A	✓	242-256	✘	N/A	N/A	✓	Twice	254-712	✓	Twice	327-712
B35	Shifting imposing date of the 1st lockdown 5 days backward + Shifting imposing date of the 2nd lockdown 1 week backward + Shifting imposing date of the 3rd lockdown 1 week backward + Two months early-launching the 1st and the 2nd dose vaccine administration + Increasing vaccination pace 100%	✓	3-73	✓	376-432	✓	463-483	✘	N/A	✘	N/A	✘	N/A	N/A	✓	Twice	254-712	✓	Twice	327-712

Table S12-1. Settings for Bangladesh's counterfactual analysis scenarios **not presented** in main text(cont'd)

Scenario setting (Mar 18, 2020--Feb 28, 2022)		Non-pharmaceutical containment measures												Medical resources	Vaccine administration Initial protocol					
		1st lockdown		2nd lockdown		3rd lockdown		4th lockdown		Added lockdown in the 2nd wave		Adding lockdown in the 5th wave			1st dose Original avg. pace: 310599/day			2nd dose Original avg. pace: 255186/day		
Scenarios	Description	Setting change	Period Day8-73	Setting change	Period Day383-432	Setting change	Period Day470-483	Setting change	Period Day492-511	Setting change	Period	Setting change	Period	Hospital capacity	Setting change	Pace change	Period Day315-712	Setting change	Pace change	Period Day386-712
BA1	Shifting imposing date of the 1st lockdown 5 days backward + shifting lifting date of the 1st lockdown 1 weeks backward	✓	3-66	✘	N/A	✘	N/A	✘	N/A	✘	N/A	✘	N/A	N/A	✘	✘	N/A	✘	✘	N/A
BA2	Shifting imposing date of the 1st lockdown 5 days backward + shifting lifting date of the 1st lockdown 1 weeks forward	✓	3-80	✘	N/A	✘	N/A	✘	N/A	✘	N/A	✘	N/A	N/A	✘	✘	N/A	✘	✘	N/A
BA3	Shifting imposing date of the 1st lockdown 5 days backward + shifting lifting date of the 1st lockdown 2 weeks forward	✓	3-87	✘	N/A	✘	N/A	✘	N/A	✘	N/A	✘	N/A	N/A	✘	✘	N/A	✘	✘	N/A
BA4	Shifting imposing date of the 2nd lockdown 1 week backward + shifting lifting date of the 2nd lockdown 1 weeks backward	✘	N/A	✓	376-425	✘	N/A	✘	N/A	✘	N/A	✘	N/A	N/A	✘	✘	N/A	✘	✘	N/A
BA5	Shifting imposing date of the 2nd lockdown 1 week backward + shifting lifting date of the 2nd lockdown 1 weeks forward	✘	N/A	✓	376-439	✘	N/A	✘	N/A	✘	N/A	✘	N/A	N/A	✘	✘	N/A	✘	✘	N/A
BA6	Shifting imposing date of the 2nd lockdown 1 week backward + shifting lifting date of the 2nd lockdown 2 weeks forward	✘	N/A	✓	376-446	✘	N/A	✘	N/A	✘	N/A	✘	N/A	N/A	✘	✘	N/A	✘	✘	N/A
BA7	Adding 1 week lockdown at the beginning of upward slope of the 2nd wave	✘	N/A	✘	N/A	✘	N/A	✘	N/A	✓	242-249	✘	N/A	N/A	✘	✘	N/A	✘	✘	N/A
BA8	Shifting imposing date of the 1st lockdown 5 days backward + Shifting lifting date of the 1st lockdown 3 weeks forward + Shifting imposing date of the 2nd lockdown 2 weeks backward + Shifting imposing date of the 3rd lockdown 2 weeks backward + Cancelling the 4th lockdown	✓	3-94	✓	369-432	✓	456-483	✓	Cancelling	✘	N/A	✘	N/A	N/A	✘	✘	N/A	✘	✘	N/A

Table S12-2. Settings for Bangladesh's counterfactual analysis scenarios **not presented** in main text

**Scenario setting** (Mar 18, 2020--Feb 28, 2022)		**Non-pharmaceutical containment measures**												Medical resources	**Vaccine administration Initial protocol**					
		1st lockdown		2nd lockdown		3rd lockdown		4th lockdown		Added lockdown in the 2nd wave		Adding lockdown in the 5th wave			1st dose Original avg. pace: 310599/day			2nd dose Original avg. pace: 255186/day		
Scenarios	Description	Setting change	Period Day8-73	Setting change	Period Day383-432	Setting change	Period Day470-483	Setting change	Period Day492-511	Setting change	Period	Setting change	Period	Hospital capacity	Setting change	Pace change	Period Day315-712	Setting change	Pace change	Period Day386-712
BA9	Shifting imposing date of the 1st lockdown 5 days backward + Shifting lifting date of the 1st lockdown 3 weeks forward + Shifting imposing date of the 2nd lockdown 2 weeks backward + Shifting imposing date of the 3rd lockdown 2 weeks backward + Adding 1 week lockdown at the beginning of upward slope of the 2nd wave	✓	3-94	✓	369-432	✓	456-483	✘	N/A	P	242-249	✘	N/A	N/A	✘	✘	N/A	✘	✘	N/A
BA10	Shifting imposing date of the 1st lockdown 5 days backward + Shifting lifting date of the 1st lockdown 3 weeks forward + Shifting imposing date of the 2nd lockdown 2 weeks backward + Shifting imposing date of the 3rd lockdown 2 weeks backward + Adding 2 weeks lockdown at the beginning of upward slope of the 2nd wave + Adding 1 week lockdown at the beginning of upward slope of the 5th wave	✓	3-94	✓	369-432	✓	456-483	✘	N/A	✓	242-256	✓	666-673	N/A	✘	Original pace	N/A	✘	✘	N/A
BA11	Increasing vaccination pace 50%	✘	N/A	✘	N/A	✘	N/A	✘	N/A	✘	N/A	✘	N/A	N/A	✓	1.5 times	N/A	✓	1.5 times	N/A
BA12	Increasing vaccination pace 80%	✘	N/A	✘	N/A	✘	N/A	✘	N/A	✘	N/A	✘	N/A	N/A	✓	1.8 times	N/A	✓	1.8 times	N/A
BA13	One month early-launching the 1st and the 2nd dose vaccine administration + Increasing vaccination pace 50%	✘	N/A	✘	N/A	✘	N/A	✘	N/A	✘	N/A	✘	N/A	N/A	✓	1.5 times	284-712	✓	1.5 times	355-712
BA14	One month early-launching the 1st and the 2nd dose vaccine administration + Increasing vaccination pace 80%	✘	N/A	✘	N/A	✘	N/A	✘	N/A	✘	N/A	✘	N/A	N/A	✓	1.8 times	284-712	✓	1.8 times	355-712
BA15	One month early-launching the 1st and the 2nd dose vaccine administration + Increasing vaccination pace 100%	✘	N/A	✘	N/A	✘	N/A	✘	N/A	✘	N/A	✘	N/A	N/A	✓	Twice	284-712	✓	Twice	355-712

Table S12-3. Settings for Bangladesh’s counterfactual analysis scenarios **not presented** in main text

**Scenario setting** (Mar 18, 2020--Feb 28, 2022)		**Non-pharmaceutical containment measures**												Medical resources	**Vaccine administration Initial protocol**					
		1st lockdown		2nd lockdown		3rd lockdown		4th lockdown		Added lockdown in the 2nd wave		Adding lockdown in the 5th wave			1st dose Original avg. pace: 310599/day			2nd dose Original avg. pace: 255186/day		
Scenarios	Description	Setting change	Period Day8-73	Setting change	Period Day383-432	Setting change	Period Day470-483	Setting change	Period Day492-511	Setting change	Period	Setting change	Period	Hospital capacity	Setting change	Pace change	Period Day315-712	Setting change	Pace change	Period Day386-712
BA16	Two months early-launching the 1st and the 2nd dose vaccine administration + Increasing vaccination pace 50%	✘	N/A	✘	N/A	✘	N/A	✘	N/A	✘	N/A	✘	N/A	N/A	✓	1.5 times	254-712	✓	1.5 times	327-712
BA17	Two months early-launching the 1st and the 2nd dose vaccine administration + Increasing vaccination pace 80%	✘	N/A	✘	N/A	✘	N/A	✘	N/A	✘	N/A	✘	N/A	N/A	✓	1.8 times	254-712	✓	1.8 times	327-712
BA18	Shifting imposing date of the 1st lockdown 5 days backward + Shifting imposing date of the 2nd lockdown 1 week backward + Shifting imposing date of the 3rd lockdown 1 week backward + Increasing vaccination pace 50%	✓	3-73	✓	376-432	✓	463-483	✘	N/A	✘	N/A	✘	N/A	N/A	✓	1.5 times	N/A	✓	1.5 times	N/A
BA19	Shifting imposing date of the 1st lockdown 5 days backward + Shifting imposing date of the 2nd lockdown 1 week backward + Shifting imposing date of the 3rd lockdown 1 week backward + Increasing vaccination pace 100%	✓	3-73	✓	376-432	✓	463-483	✘	N/A	✘	N/A	✘	N/A	N/A	✓	Twice	N/A	✓	Twice	N/A
BA20	Shifting imposing date of the 1st lockdown 5 days backward + Shifting lifting date of the 1st lockdown 3 weeks forward + Shifting imposing date of the 2nd lockdown 2 weeks backward + Shifting imposing date of the 3rd lockdown 2 weeks backward + Cancelling the 4th lockdown + Increasing vaccination pace 100%	✓	3-94	✓	369-432	✓	456-483	✓	Cancelling	✘	N/A	✘	N/A	N/A	✓	Twice	N/A	✓	Twice	N/A

Table S12-4. Settings for Bangladesh's counterfactual analysis scenarios **not presented** in main text

**Scenario setting** (Mar 18, 2020--Feb 28, 2022)		**Non-pharmaceutical containment measures**												Medical resources	**Vaccine administration Initial protocol**					
		1st lockdown		2nd lockdown		3rd lockdown		4th lockdown		Added lockdown in the 2nd wave		Adding lockdown in the 5th wave			1st dose Original avg. pace: 310599/day			2nd dose Original avg. pace: 255186/day		
Scenarios	Description	Setting change	Period Day8-73	Setting change	Period Day383-432	Setting change	Period Day470-483	Setting change	Period Day492-511	Setting change	Period	Setting change	Period	Hospital capacity	Setting change	Pace change	Period Day315-712	Setting change	Pace change	Period Day386-712
BA21	Shifting imposing date of the 1st lockdown 5 days backward + Shifting lifting date of the 1st lockdown 3 weeks forward + Shifting imposing date of the 2nd lockdown 2 weeks backward + Shifting imposing date of the 3rd lockdown 2 weeks backward + Increasing vaccination pace 100%	✓	3-94	✓	369-432	✓	456-483	✘	N/A	✘	N/A	✘	N/A	N/A	✓	Twice	N/A	✓	Twice	N/A
BA22	Shifting imposing date of the 1st lockdown 5 days backward + Shifting lifting date of the 1st lockdown 3 weeks forward + Shifting imposing date of the 2nd lockdown 2 weeks backward + Shifting imposing date of the 3rd lockdown 2 weeks backward + Adding 2 weeks lockdown at the beginning of upward slope of the 2nd wave + Increasing vaccination pace 100%	✓	3-94	✓	369-432	✓	456-483	✘	N/A	✓	242-256	✘	N/A	N/A	✓	Twice	N/A	✓	Twice	N/A
BA23	Shifting imposing date of the 1st lockdown 5 days backward + Shifting lifting date of the 1st lockdown 3 weeks forward + Shifting imposing date of the 2nd lockdown 2 weeks backward + Shifting imposing date of the 3rd lockdown 2 weeks backward + Adding 2 weeks lockdown at the beginning of upward slope of the 2nd wave + One month early-launching the 1st and the 2nd dose vaccine administration + Increasing vaccination pace 50%	✓	3-94	✓	369-432	✓	456-483	✘	N/A	✓	242-256	✘	N/A	N/A	✓	1.5 times	284-712	✓	1.5 times	355-712

Table S12-5. Settings for Bangladesh's counterfactual analysis scenarios **not presented** in main text

**Scenario setting** (Mar 18, 2020--Feb 28, 2022)		**Non-pharmaceutical containment measures**												**Medical resources**	**Vaccine administration Initial protocol**					
		1st lockdown		2nd lockdown		3rd lockdown		4th lockdown		Added lockdown in the 2nd wave		Adding lockdown in the 5th wave			1st dose Original avg. pace: 310599/day			2nd dose Original avg. pace: 255186/day		
Scenarios	Description	Setting change	Period Day8-73	Setting change	Period Day383-432	Setting change	Period Day470-483	Setting change	Period Day492-511	Setting change	Period	Setting change	Period	Hospital capacity	Setting change	Pace change	Period Day315-712	Setting change	Pace change	Period Day386-712
BA24	Shifting imposing date of the 1st lockdown 5 days backward + Shifting lifting date of the 1st lockdown 3 weeks forward + Shifting imposing date of the 2nd lockdown 2 weeks backward + Shifting imposing date of the 3rd lockdown 2 weeks backward + Adding 2 weeks lockdown at the beginning of upward slope of the 2nd wave + Two months early-launching the 1st and the 2nd dose vaccine administration + Increasing vaccination pace 50%	✓	3-94	✓	369-432	✓	456-483	✘	N/A	✓	242-256	✘	N/A	N/A	✓	1.5 times	254-712	✓	1.5 times	327-712
BA25	Shifting imposing date of the 1st lockdown 5 days backward + Shifting lifting date of the 1st lockdown 3 weeks forward + Shifting imposing date of the 2nd lockdown 2 weeks backward + Shifting imposing date of the 3rd lockdown 2 weeks backward + Two months early-launching the 1st and the 2nd dose vaccine administration + Increasing vaccination pace 100%	✓	3-94	✓	369-432	✓	456-483	✘	N/A	✘	N/A	✘	N/A	N/A	✓	Twice	254-712	✓	Twice	327-712

S4 Simulation Results

Pakistan Bangladesh

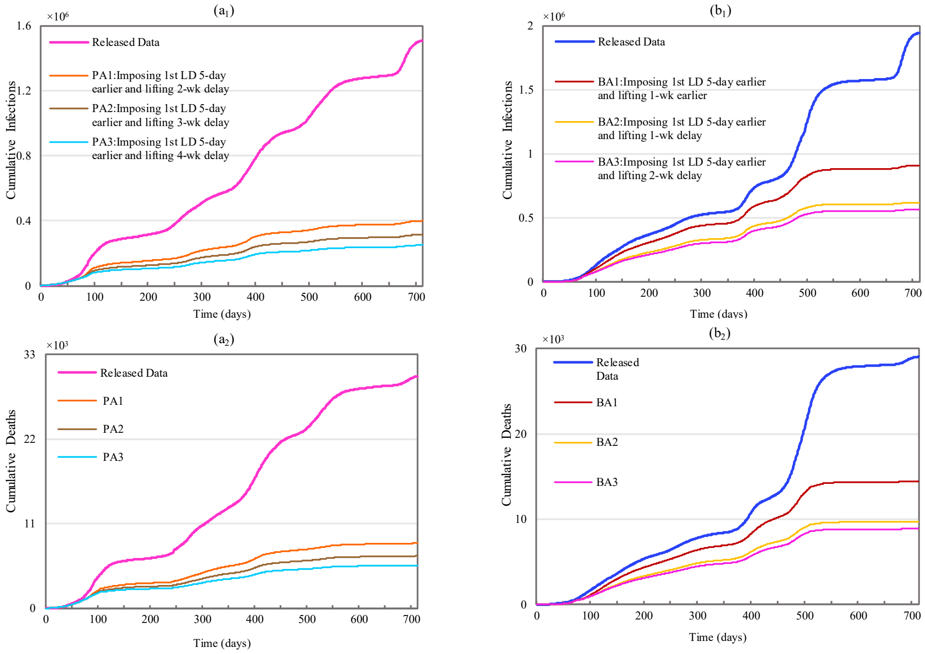

Figure S3. Effects of changing timing and duration first lockdown on the cumulative infections and cumulative deaths in **Pakistan** and **Bangladesh** (Scenario **not presented** in main text)

Pakistan Bangladesh

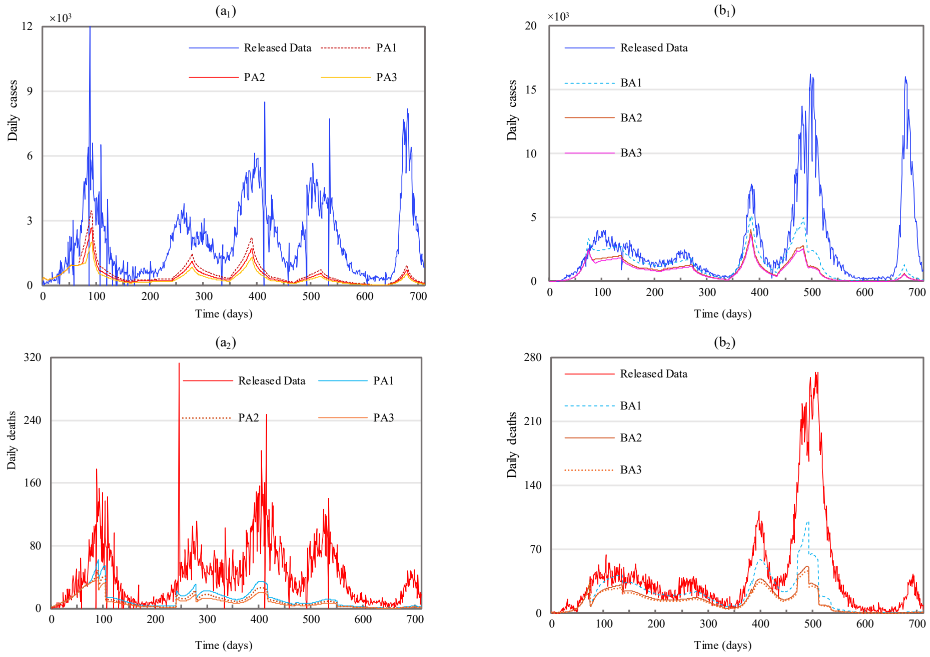

Figure S4. Impact of changing 1st lockdown in **Pakistan** and **Bangladesh** -daily infections and deaths (For scenarios **not presented** in main text)

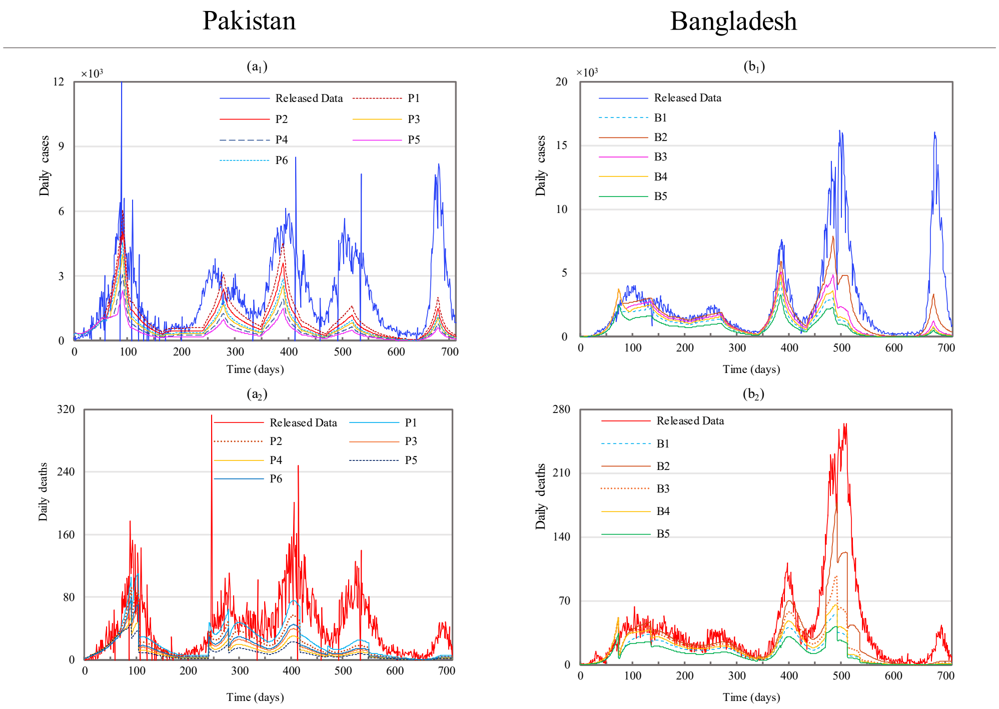

Figure S5. Impact of changing 1st lockdown in **Pakistan** and **Bangladesh** -daily infections and deaths (For scenarios **presented** in main text)

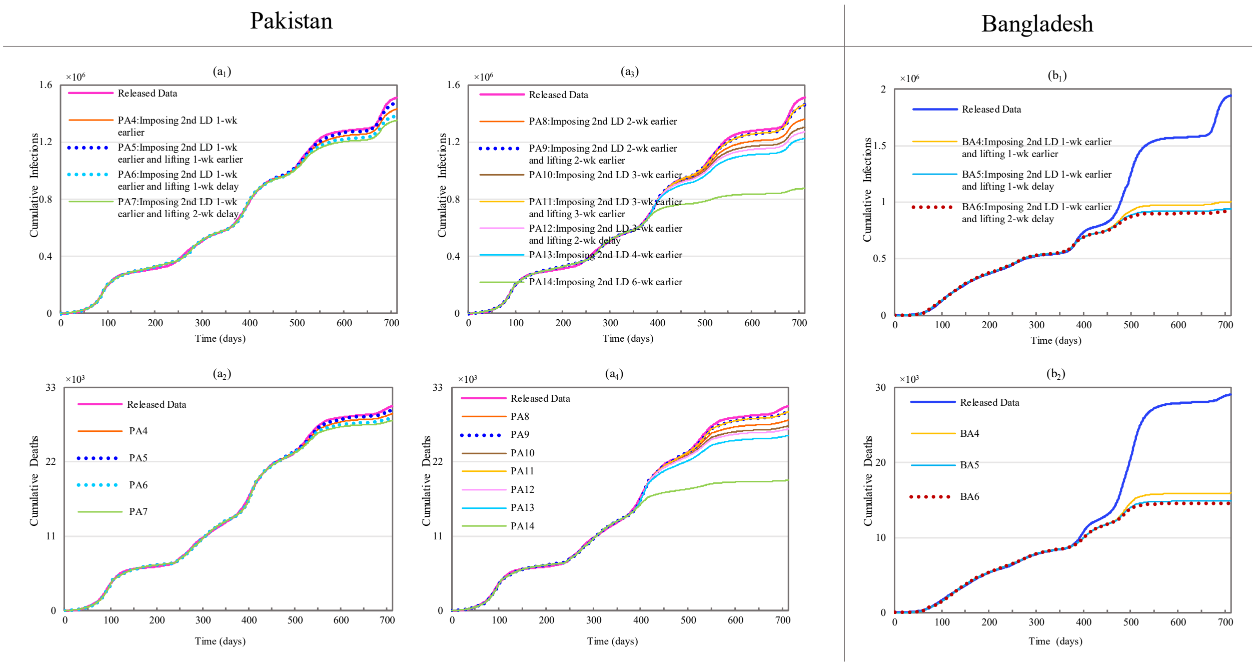

Figure S6. Impacts of changing timing of 2nd in Pakistan and 2nd and 3rd lockdowns on the cumulative infections and cumulative deaths in Bangladesh (Scenario **not presented** in main text)

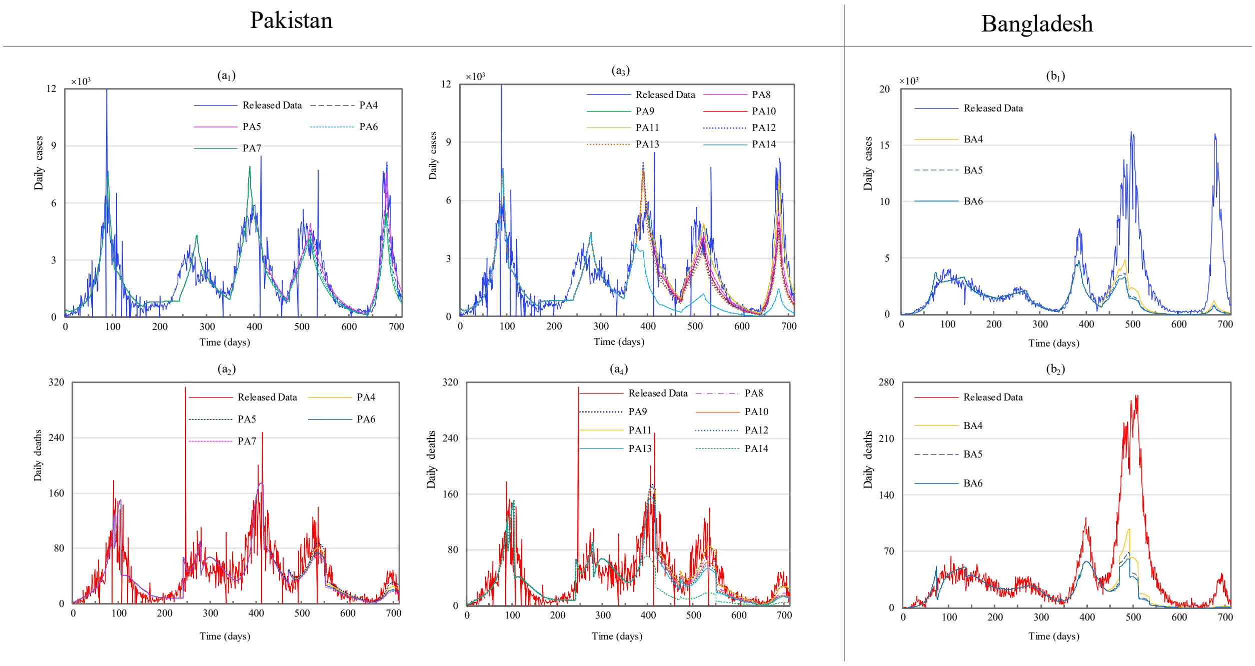

Figure S7. Impacts of changing timing of 2nd in **Pakistan** and 2nd and 3rd lockdowns in **Bangladesh** -daily infections and deaths (For scenarios **not presented** in main text)

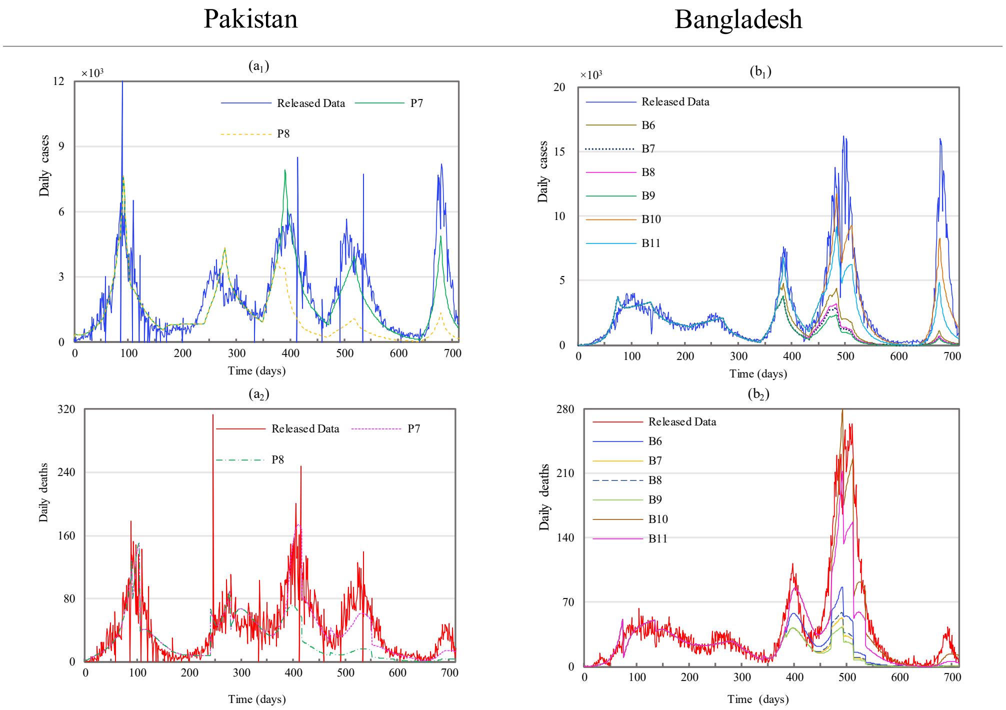

Figure S8. Impacts of changing timing of 2nd in **Pakistan** and 2nd and 3rd lockdowns in **Bangladesh** -daily infections and deaths (For scenarios **presented** in main text)

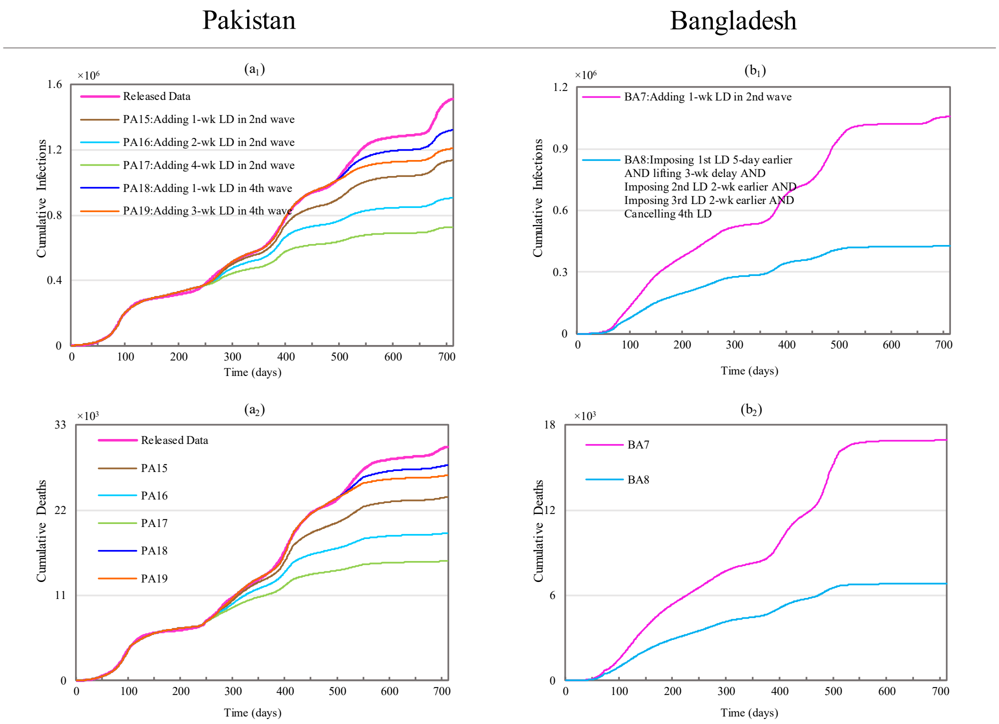

Figure S9. Effects of adding lockdown on the cumulative infections and cumulative deaths in **Pakistan** and **Bangladesh** (Scenario **not presented** in main text)

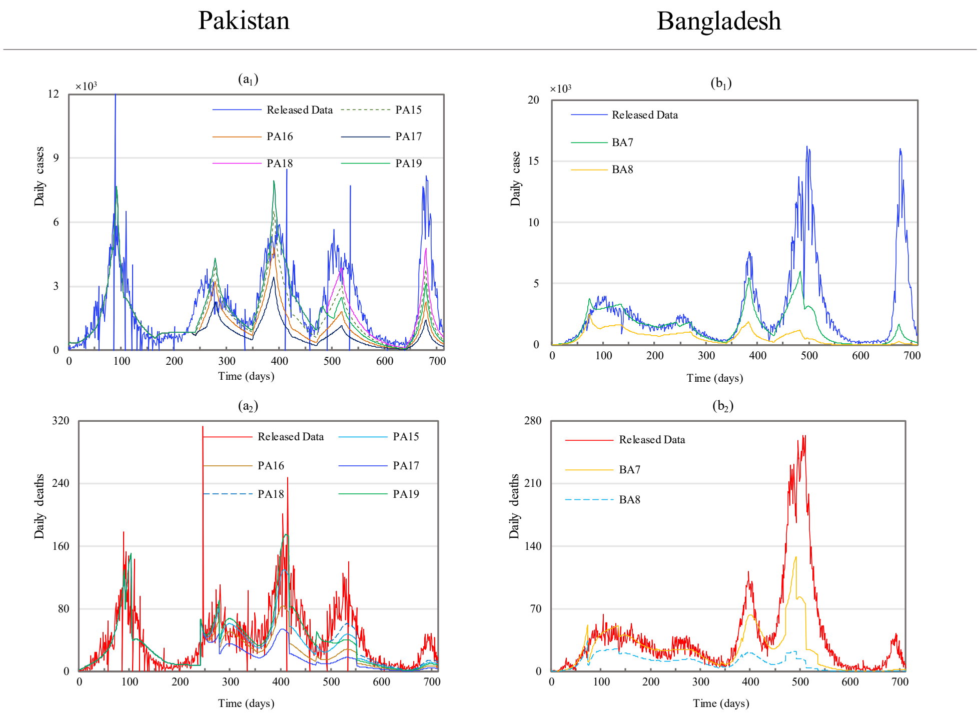

Figure S10. Effects of adding lockdown in **Pakistan** and **Bangladesh** -daily infections and deaths (For scenarios **not presented** in main text)

Pakistan Bangladesh

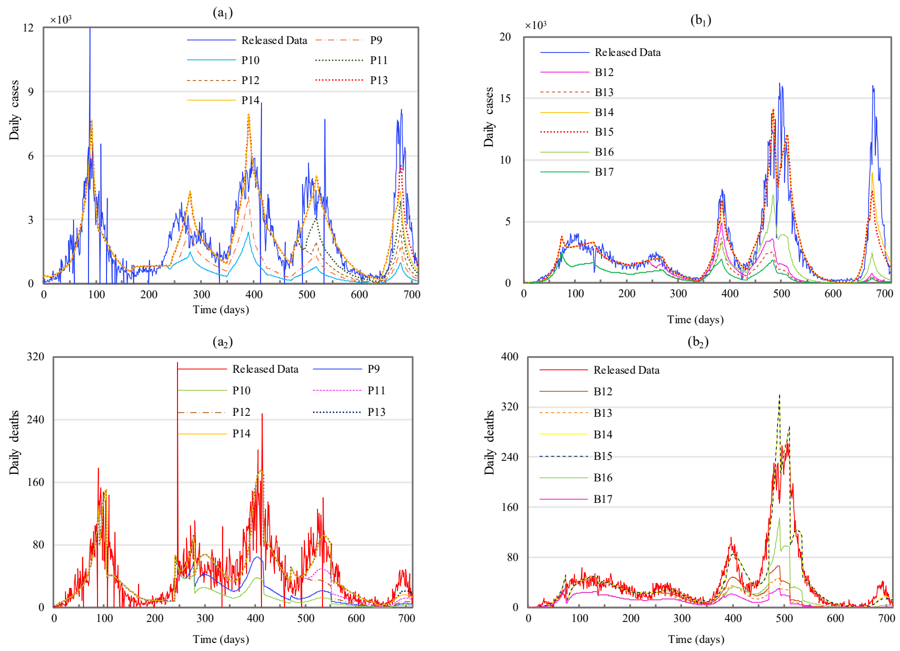

Figure S11. Effects of adding lockdown in **Pakistan** and **Bangladesh** -daily infections and deaths (For scenarios **presented** in main text)

Pakistan Bangladesh

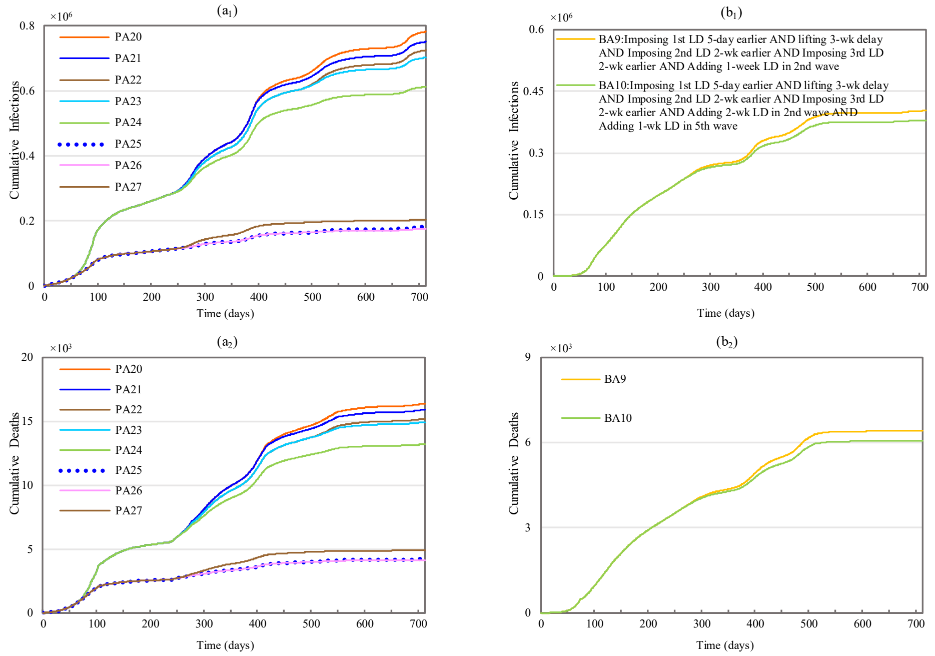

Figure S12. Effects of simultaneously changing multiple lockdowns on the cumulative infections and cumulative deaths in **Pakistan** and **Bangladesh** (Scenario **not presented** in main text)

Pakistan Bangladesh

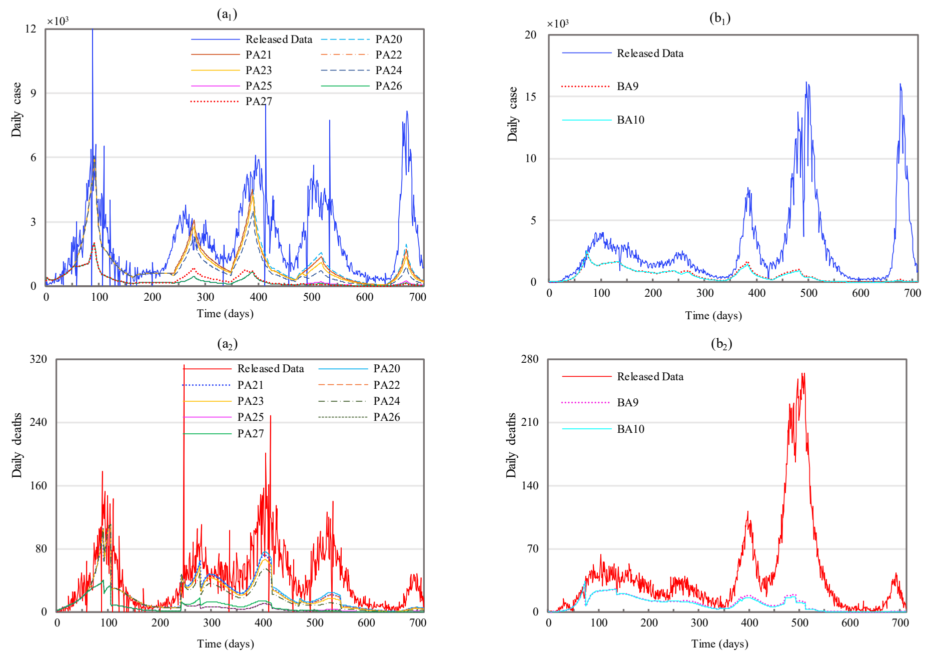

Figure S13. Effects of simultaneously changing multiple lockdowns in **Pakistan** and **Bangladesh** -daily infections and deaths (For scenarios **not presented** in main text)

Pakistan Bangladesh

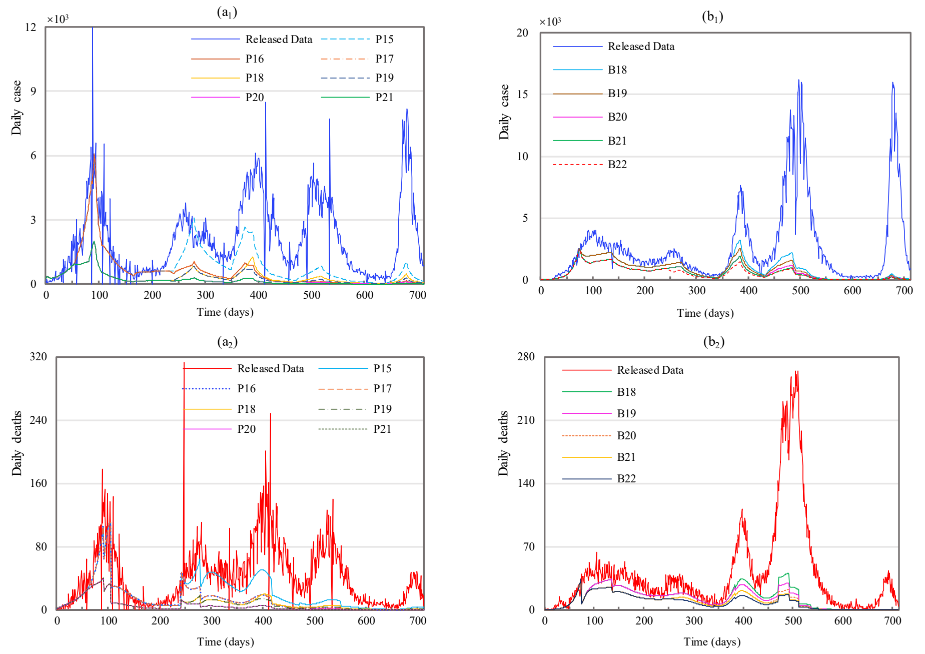

Figure S14. Effects of simultaneously changing multiple lockdowns in **Pakistan** and **Bangladesh** -daily infections and deaths (For scenarios **presented** in main text)

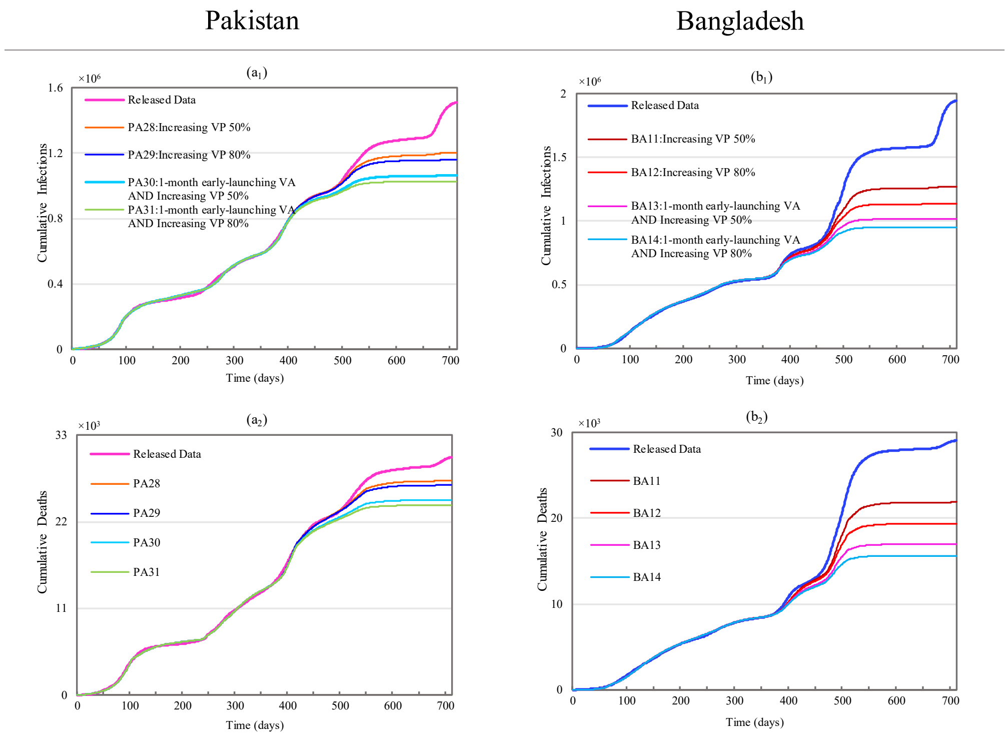

Figure S15. Effects of changing hospital capacity and changing lockdowns on the cumulative infections and cumulative deaths in **Pakistan** and **Bangladesh** (Scenario **not presented** in main text)

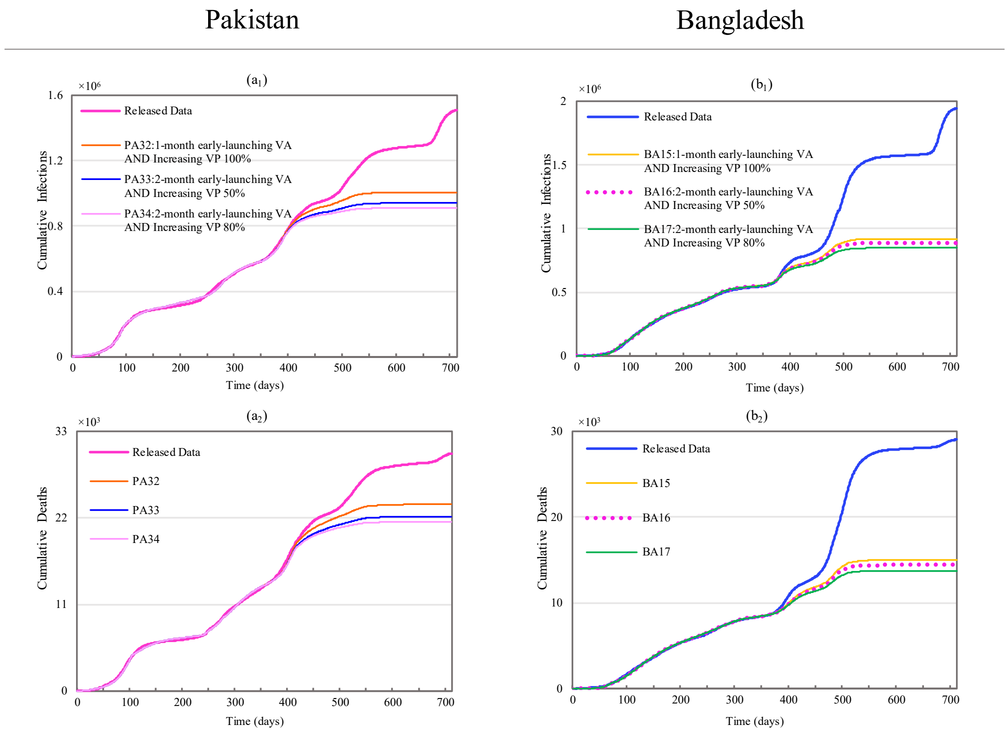

Figure S16. Effects of changing vaccination level on the cumulative infections and cumulative deaths in **Pakistan** and **Bangladesh** (Scenario **not presented** in main text)

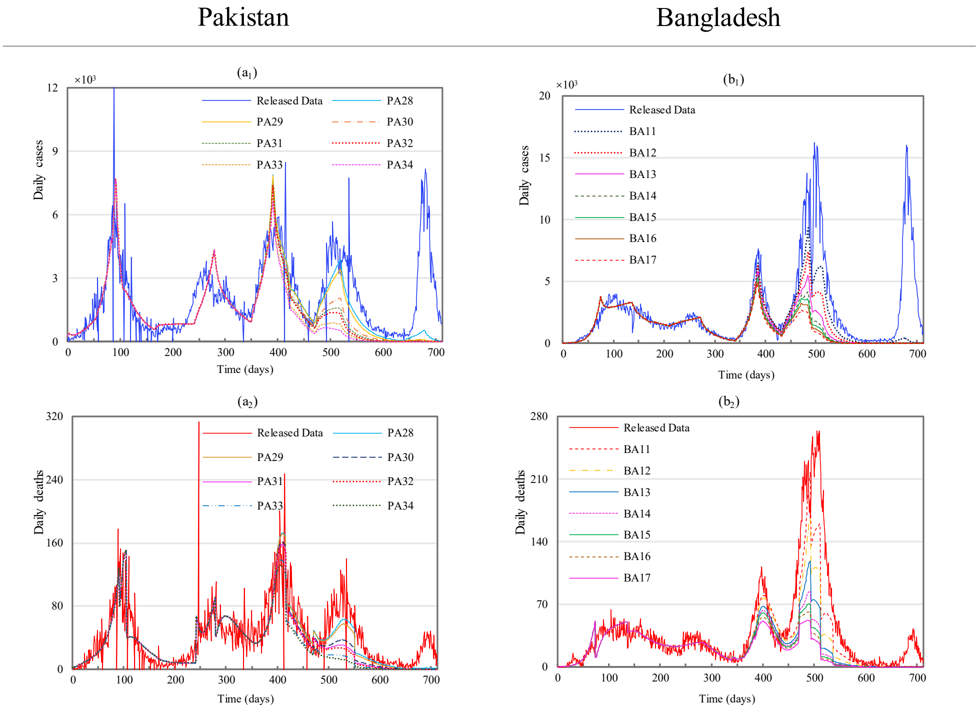

Figure S17. Effects of changing vaccination level on the cumulative infections and cumulative deaths in **Pakistan** and **Bangladesh** -daily infections and deaths (For scenarios **not presented** in main text)

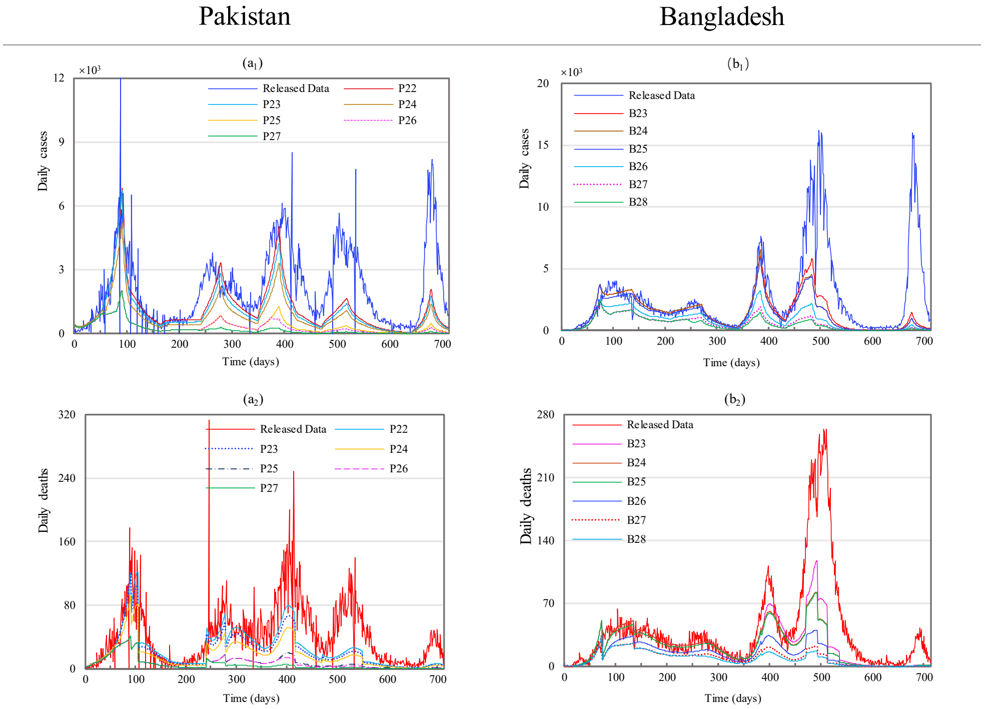

Figure S18. Effects of changing hospital capacity and changing lockdowns in **Pakistan** and **Bangladesh** -daily infections and deaths (For scenarios **presented** in main text)

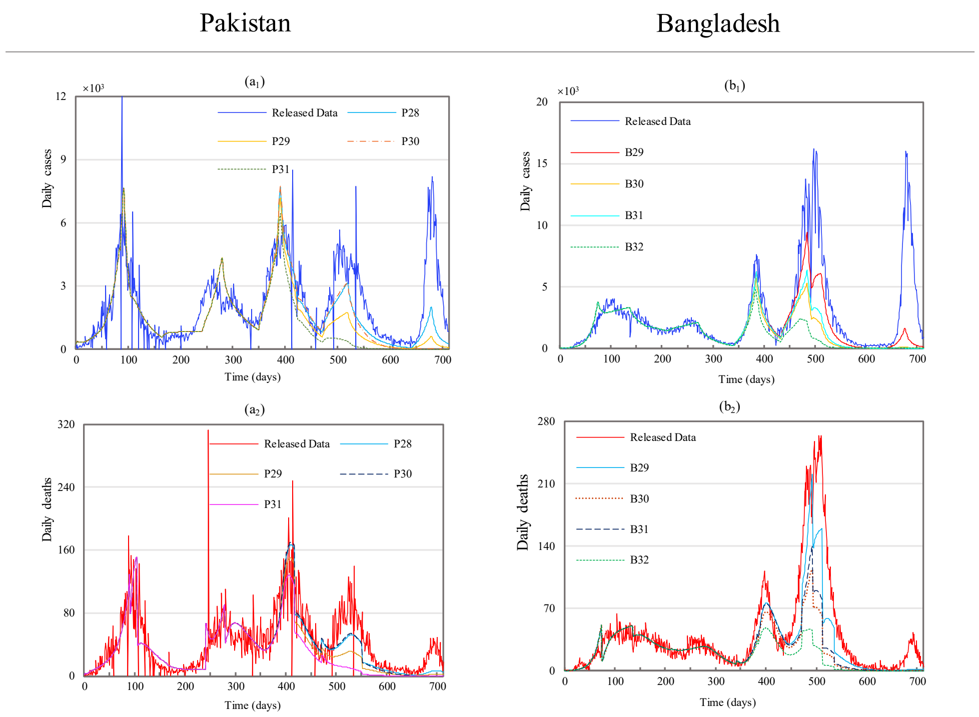

Figure S19. Effects of changing vaccination level on the cumulative infections and cumulative deaths in **Pakistan** and **Bangladesh** -daily infections and deaths (For scenarios **presented** in main text)

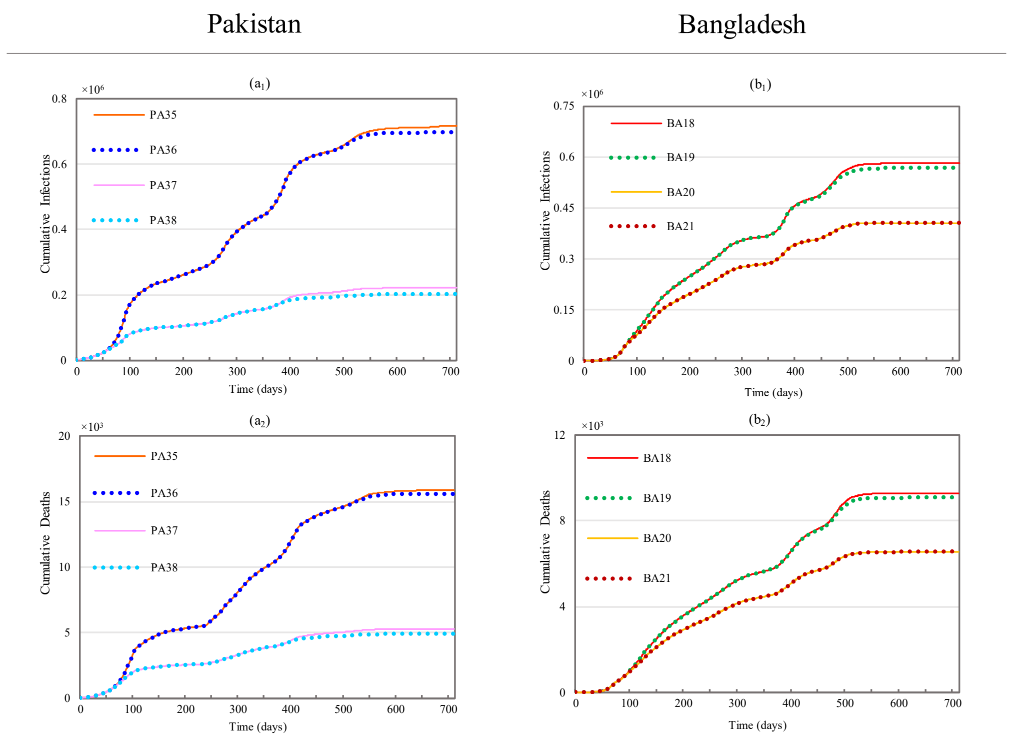

Figure S20-1. Effects of simultaneously resetting lockdown, hospital capacity, and vaccination level on the cumulative infections and cumulative deaths in **Pakistan** and **Bangladesh** (Scenario **not presented** in main text)

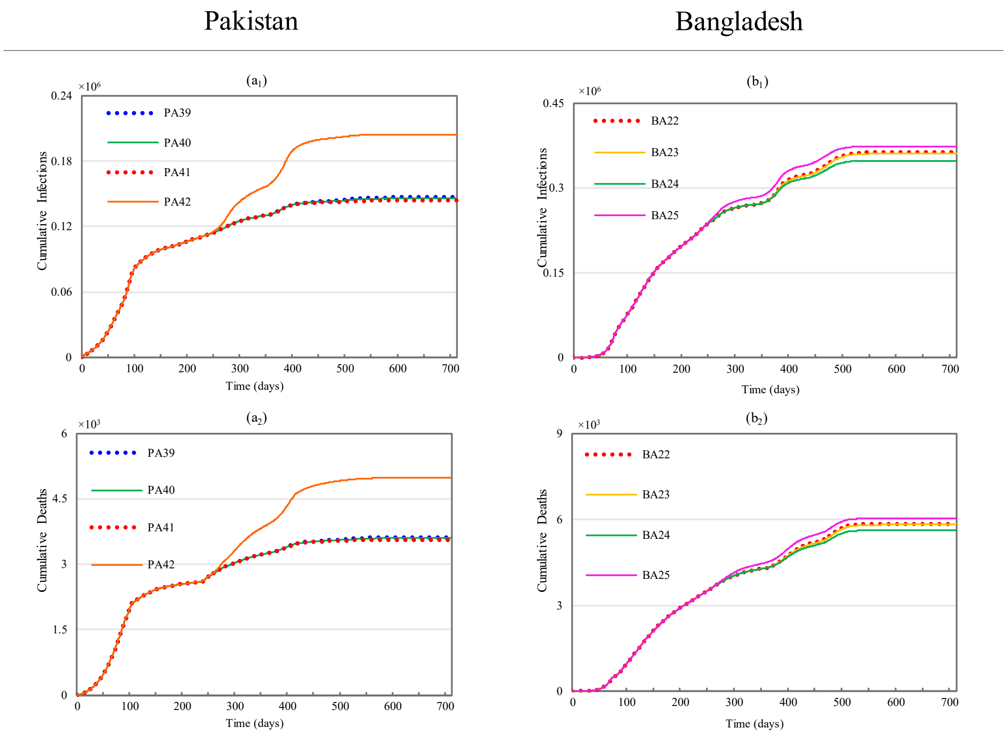

Figure S20-2. Effects of simultaneously resetting lockdown, hospital capacity, and vaccination level on the cumulative infections and cumulative deaths in **Pakistan** and **Bangladesh** (Scenario **not presented** in main text)

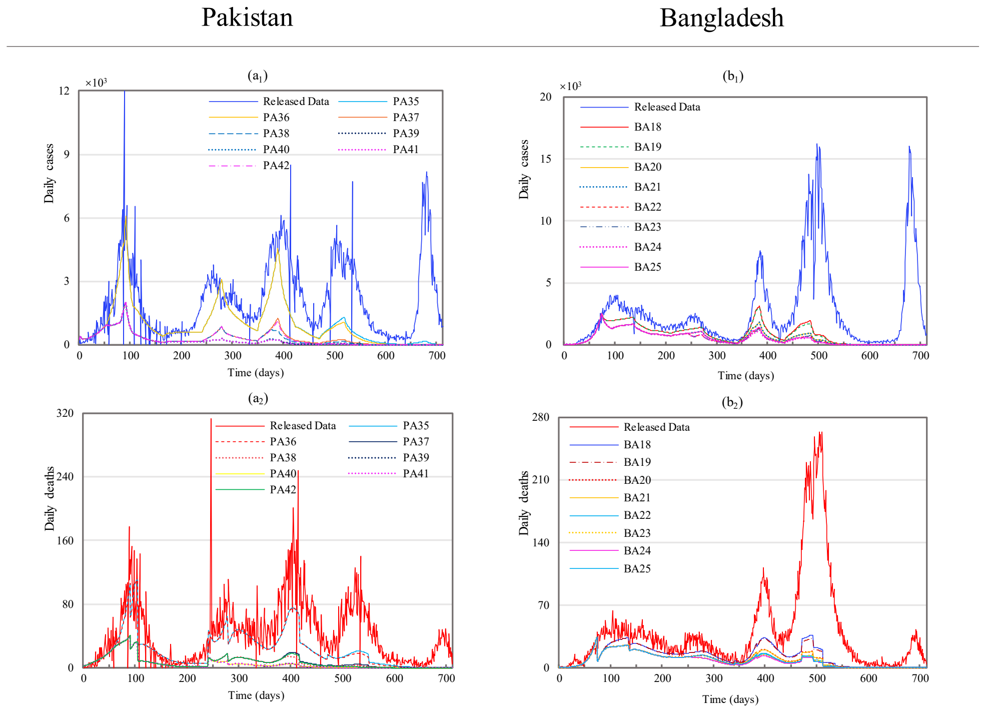

Figure S21. Effects of simultaneously changing lockdown, hospital capacity, and vaccination level in **Pakistan** and **Bangladesh** -daily infections and deaths (For scenarios **not presented** in main text)

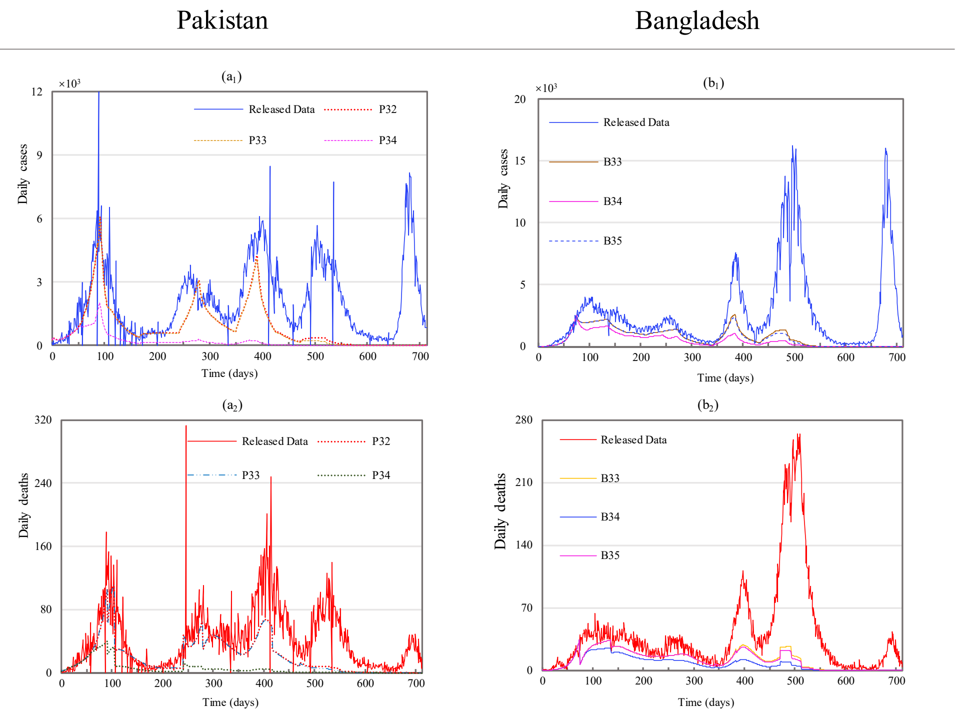

Figure S22. Effects of simultaneously resetting lockdown, hospital capacity, and vaccination level in **Pakistan** and **Bangladesh** -daily infections and deaths (For scenarios **presented** in main text)

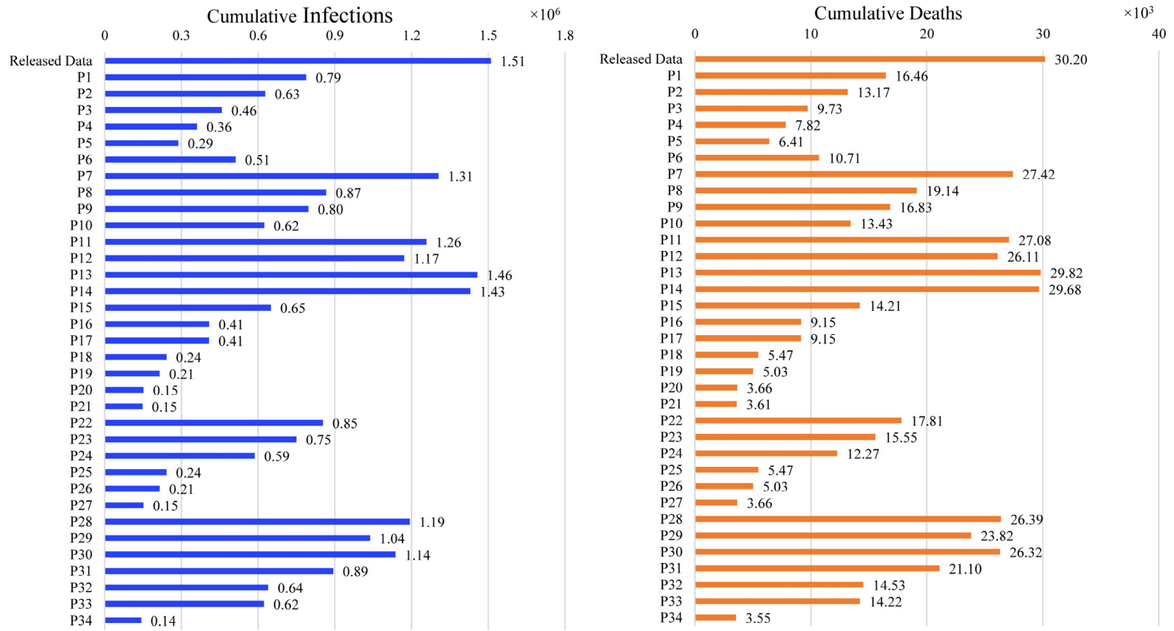

Figure S23. Results for all scenarios in counterfactual analysis **presented** in main text - **Pakistan**

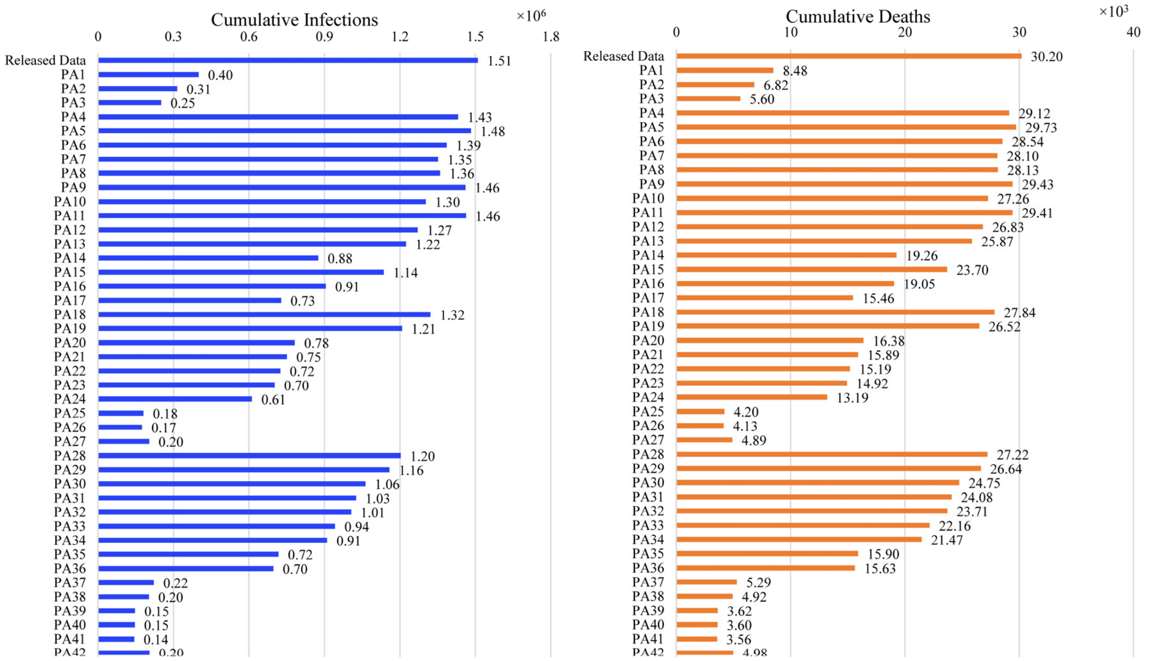

Figure S24. Results for scenarios in counterfactual analysis **not presented** in main text - **Pakistan**

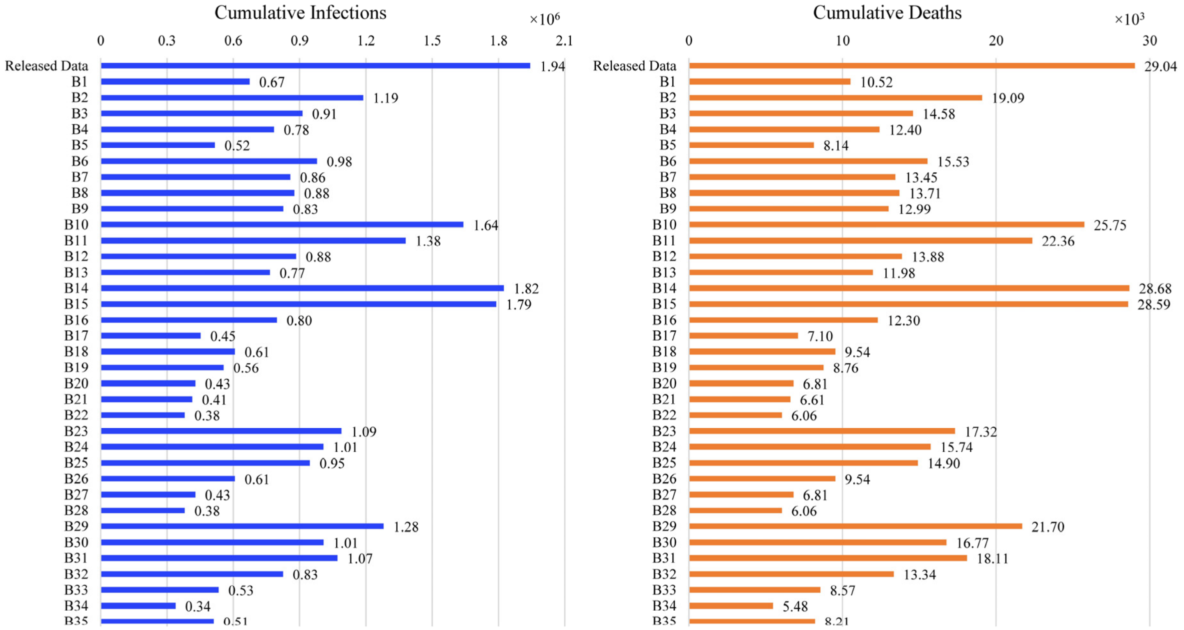

Figure S25. Results for all scenarios in counterfactual analysis **presented** in main text - **Bangladesh**

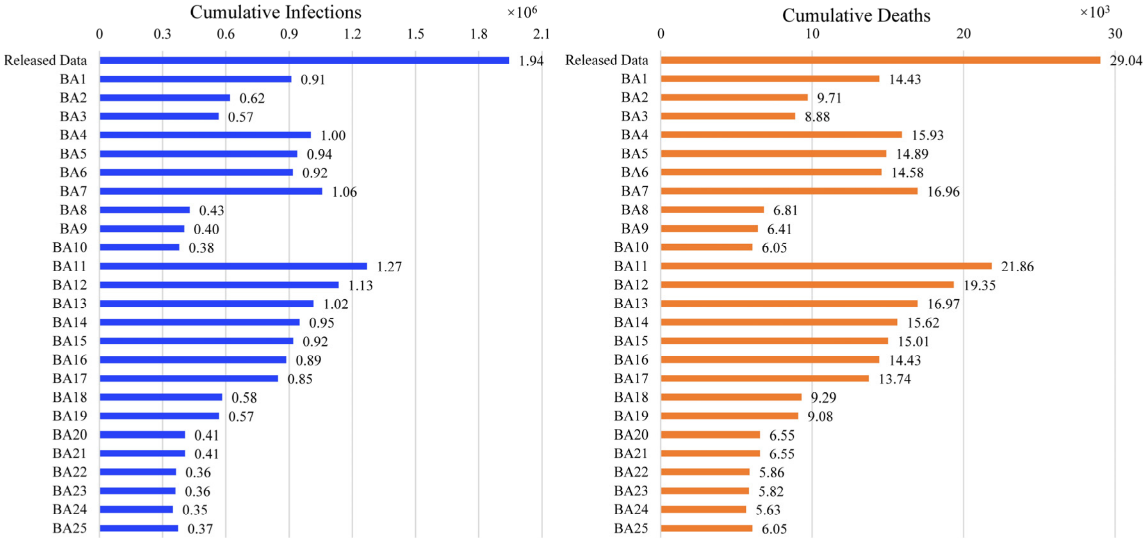

Figure S26. Results for scenarios in counterfactual analysis **not presented** in main text – **Bangladesh**
